# IL-33 stimulates the anticancer activities of eosinophils through extracellular vesicle-driven reprogramming of tumor cells

**DOI:** 10.1186/s13046-024-03129-1

**Published:** 2024-07-27

**Authors:** Adriana Rosa Gambardella, Caterina Antonucci, Cristiana Zanetti, Francesco Noto, Sara Andreone, Davide Vacca, Valentina Pellerito, Chiara Sicignano, Giuseppe Parrottino, Valentina Tirelli, Antonella Tinari, Mario Falchi, Adele De Ninno, Luca Businaro, Stefania Loffredo, Gilda Varricchi, Claudio Tripodo, Claudia Afferni, Isabella Parolini, Fabrizio Mattei, Giovanna Schiavoni

**Affiliations:** 1 Department of Oncology and Molecular Medicine, Istituto Superiore Di Sanità, Rome, Italy; 2 Tumor Immunology Unit, Department of Sciences for Health Promotion and Mother-Child Care ”G. D’Alessandro”, University of Palermo, Palermo, 90127 Italy; 3 Core Facilities, Istituto Superiore Di Sanità, Rome, Italy; 4 National Center for Gender Medicine, Istituto Superiore Di Sanità, Rome, Italy; 5 National AIDS Center, Istituto Superiore Di Sanità, Rome, Italy; 6 CNR-IFN Institute for Photonics and Nanotechnologies, Rome, Italy; 7 Department of Translational Medical Sciences, University of Naples Federico II, Naples, Italy; 8 World Allergy Organization (WAO), Center of Excellence (CoE), Naples, 80131 Italy; 9 Institute of Experimental Endocrinology and Oncology, National Research Council (CNR), Naples, 80131 Italy; 10 National Center for Drug Research and Evaluation, Istituto Superiore Di Sanità, Rome, Italy; 11 Laboratory of Molecular Medicine and DNA Repair, Department of Medicine, University of Udine, Udine, Italy

**Keywords:** Extracellular vesicles, Cancer, Eosinophils, IL-33, Tumor microenvironment, Epithelial to mesenchymal transition, Cell proliferation, RNA sequencing

## Abstract

Immune cell-derived extracellular vesicles (EV) affect tumor progression and hold promise for therapeutic applications. Eosinophils are major effectors in Th2-related pathologies recently implied in cancer. Here, we evaluated the anti-tumor activities of eosinophil-derived EV following activation with the alarmin IL-33. We demonstrate that IL-33-activated mouse and human eosinophils produce higher quantities of EV with respect to eosinophils stimulated with IL-5. Following incorporation of EV from IL-33-activated eosinophils (Eo33-EV), but not EV from IL-5-treated eosinophils (Eo5-EV), mouse and human tumor cells increased the expression of cyclin-dependent kinase inhibitor (CDKI)-related genes resulting in cell cycle arrest in G0/G1, reduced proliferation and inhibited tumor spheroid formation. Moreover, tumor cells incorporating Eo33-EV acquired an epithelial-like phenotype characterized by E-Cadherin up-regulation, N-Cadherin downregulation, reduced cell elongation and migratory extent in vitro, and impaired capacity to metastasize to lungs when injected in syngeneic mice. RNA sequencing revealed distinct mRNA signatures in Eo33-EV and Eo5-EV with increased presence of tumor suppressor genes and enrichment in pathways related to epithelial phenotypes and negative regulation of cellular processes in Eo33-EV compared to Eo5-EV. Our studies underscore novel IL-33-stimulated anticancer activities of eosinophils through EV-mediated reprogramming of tumor cells opening perspectives on the use of eosinophil-derived EV in cancer therapy.

**Supplementary Information:**

The online version contains supplementary material available at 10.1186/s13046-024-03129-1.

## Introduction

Extracellular vesicles (EV), including exosomes, play a crucial role in cancer progression through the transfer of a broad spectrum of signaling molecules between tumor, immune infiltrating and stromal cells within the tumor microenvironment (TME) [1]. EV deliver a cargo of functional proteins, lipids and nucleic acids (mRNA, lncRNA and miRNA) that reflects the functional status of the producing cell [2] and induce downstream signaling cascades on receiving cells shaping their phenotype and functions [3]. The primary mechanism of EV entry into target cells is endocytosis that mediates the internalization and transfer of EV cargo for rapid translation and modulation of gene expression [3, 4]. In the TME, EV secreted by immune cells can inhibit or promote tumor progression, depending on the producing cell type and status, the carried molecules and the phenotypic modifications induced in cancer and other cells in the TME [5]. For example, EV derived from classically activated (M1) and alternatively activated (M2) macrophages respectively inhibit [6, 7] and promote [8–11] tumor proliferation, invasion and metastasis through delivery of distinct spectra of miRNAs and lncRNAs. EV secreted by NK cells carry cytotoxic proteins (granzymes, granulysin and perforin) and express surface molecules (FasL, DNAM-I) that trigger tumor cell apoptosis. Moreover, miRNAs (MiR-186 and MiR-3607-3p) in NK-derived EV inhibit cancer cell proliferation, migration and invasion [12]. EV released by T cells [13–15], B cells [16], dendritic cells [17] and mast cells [18] can also target cancer cells variably affecting tumor progression. The role of eosinophil EV in the TME is currently unknown.

Eosinophils are important components of the TME where they play diverse roles in regulating tumor progression [19]. Eosinophils secrete several soluble mediators, including cytokines, chemokines, angiogenic and growth factors that variably affect immune responses and tumor progression [19, 20]. Through degranulation, eosinophils release a plethora of cytotoxic mediators, such as cationic proteins and granzymes that induce the killing of mouse [21–24] and human [25–27] tumor cells. This effector function of eosinophils can be augmented by activation stimuli, such as IFN-γ [24] and IL-33 [21, 22]. In particular, IL-33 can stimulate eosinophil-dependent anti-tumor immunity in various tumor models [22, 23, 28]. Eosinophils secrete EV following activation with IFN-γ and the release of EV is higher in eosinophils from asthmatic subjects compared to healthy individuals [29]. Moreover, EV from eosinophils of asthmatics activate in an autocrine manner eosinophils [30] and participate in airway remodeling by affecting inflammation-related gene expression in structural lung cells [31]. These observations demonstrate the potential of eosinophil-derived EV to reprogram and shape target cells.

In the present study, we have investigated the impact of IL-33 activation on EV release by mouse and human eosinophils and the effects of eosinophil-derived EV in shaping the phenotype of tumor cells. We show that IL-33 both stimulates the secretion and qualitatively affects the molecular cargo of EV released by eosinophils. Following incorporation into target tumor cells, EV from IL-33-activated eosinophils transcriptionally reprogram tumor cells to inhibit cancer proliferation and malignant progression. Our results provide the first evidence of a role for eosinophil-secreted EV in cancer progression.

## Results

### IL-33 stimulates EV secretion by eosinophils

**Fig. 1:**
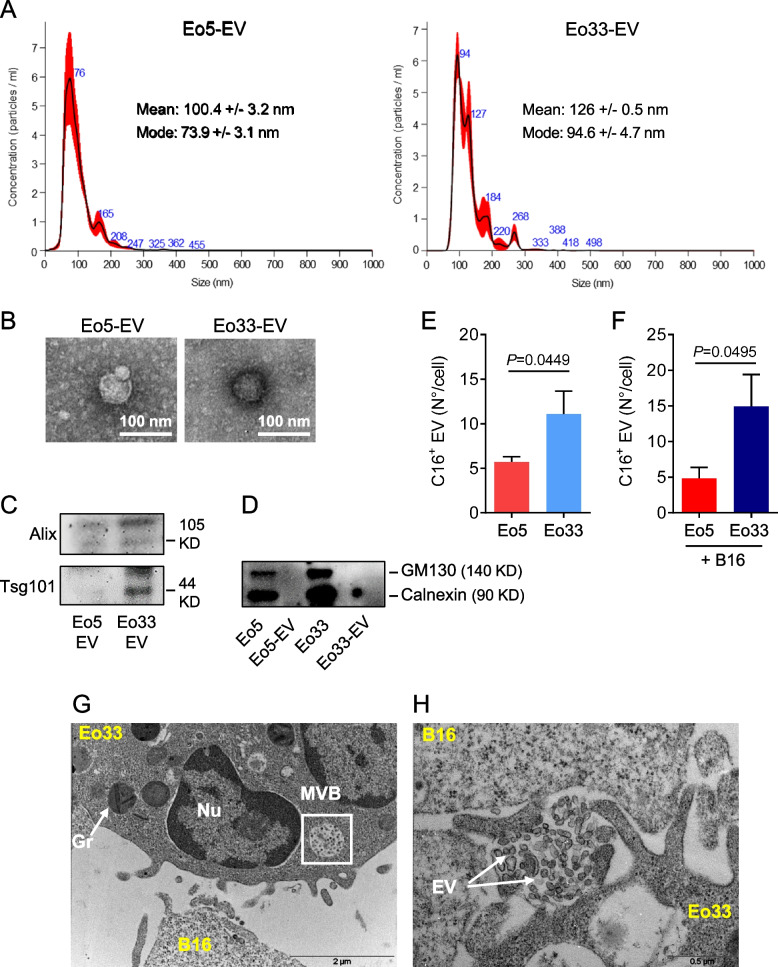
IL-33 stimulates the release of EV by eosinophils. EV were isolated from Eo5 and Eo33 conditioned media by serial ultracentrifugations. **A** NTA analysis of vesicle size and (**B**) transmission electron microscopy of negative stained Eo5-EV and Eo33-EV. Western blotting for (**C**) EV markers Alix and Tsg101 in Eo5-EV and Eo33-EV and (**D**) GM130 and Calnexin in Eo5-EV, Eo33-EV and their producing cells. Flow cytometry quantification of fluorescent EV released by Bodipy FL-C16 labelled Eo5 and Eo33 (**E**) cultured alone or (**F**) in the presence of B16.F10 melanoma cells (20:1 ratio) for 24 h. Data are expressed as number of EV released per cell. Mean (SD) of four (**E**) and three (**F**) separate experiments is shown. **G-H** Transmission electron microscopy analysis of Eo33 co-cultured with B16.F10 melanoma cells (10:1 ratio) for 1 h, showing the presence of multivesicular bodies (MVB, **G**) and EV release (**H**) by an eosinophil in proximity of a tumor cell. Gr: granule. Nu: nucleus

We previously reported that mouse bone marrow-derived eosinophils, obtained by culturing bone marrow progenitors in presence of IL-5, undergo phenotypic and functional activation following exposure to IL-33 [21, 22]. We asked whether IL-33 stimulation could also affect EV secretion by these eosinophils. To this end, we isolated and characterized EV from the culture medium of IL-33 activated (Eo33) and control IL-5 stimulated (Eo5) mouse bone marrow-derived eosinophils by serial ultracentrifugations. Nanoparticle tracking analysis (NTA) showed the size of the mainly represented vesicle population released by Eo5 (mode 73.9 nm) and Eo33 (mode 94.6 nm) (Fig. 1A) and excluded the presence of lytic granules (500–1000 nm) [32] in the population. Negative staining transmission electron microscopy showed typical ultrastructure and integrity of EV released by Eo5 and Eo33 (Fig. 1B). Western blotting analysis confirmed protein expression of EV markers Tsg101 and Alix, which were present at increased levels in Eo33-derived EV as compared to Eo5-EV (Fig. 1C) and absence of GM130 and Calnexin (Fig. 1D), indicating absence of contaminating organelles. To evaluate whether IL-33 stimulated the release of EV by eosinophils, we generated fluorescent EV by Bodipy FL C16 metabolic labelling of eosinophils and quantified them by flow cytometry [33, 34]. The results show that Eo33 secreted significantly higher numbers (~twofold) of fluorescent (C16^+^) EV with respect to Eo5 (Fig. 1E). Moreover, when FL C16-labelled eosinophils where co-cultured with B16.F10 melanoma cells we also observed a ~twofold higher EV secretion by Eo33 with respect to Eo5 (Fig. 1F). Transmission electron microscopy of IL-33 activated eosinophils co-cultured with B16.F10 melanoma cells revealed the presence of multivesicular bodies (MVB; Fig. 1G) and release of EV (Fig. 1H) in eosinophils upon contact with target tumor cells. These results indicate that activation with IL-33 stimulates the secretion of EV by eosinophils and that this process occurred also in proximity of tumor cells.

### Eosinophil-derived EV are incorporated by tumor cells

**Fig. 2:**
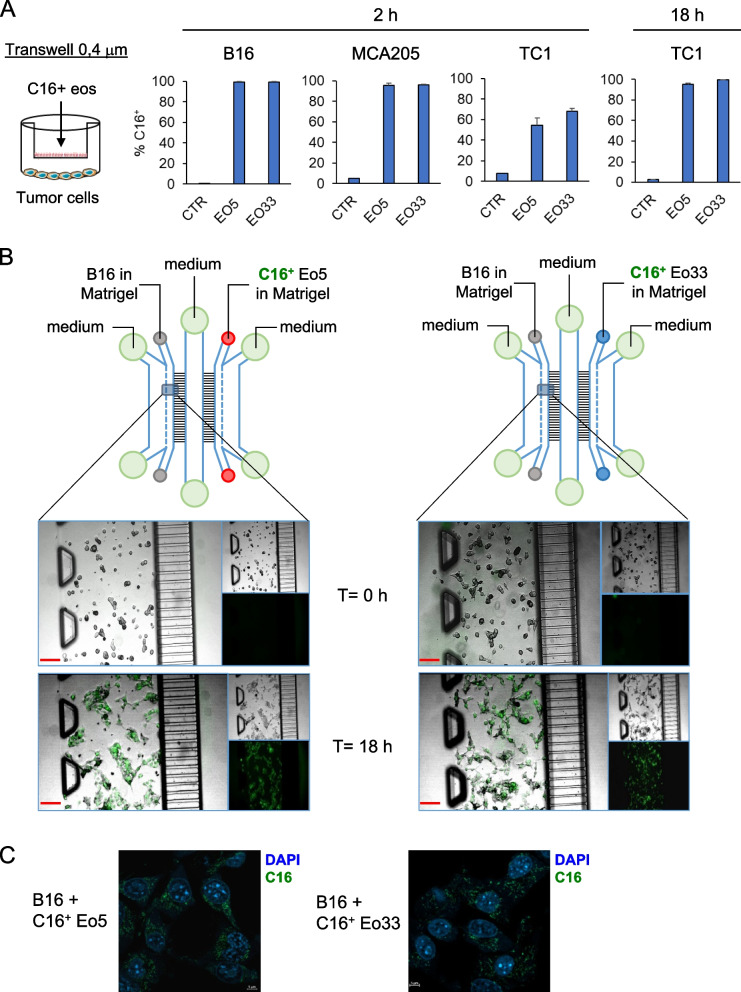
Transfer of eosinophil-derived EV to tumor cells. **A** Eo5 and Eo33 were labelled with Bodipy FL-C16 and then co-cultured with tumor cells separated by a 0.4 μm pore sized insert. C16^+^ EV intake by tumor cells after the indicated culture times, revealed by flow cytometry analysis of green fluorescence acquisition. CTR: tumor cells cultured alone. Mean (SD) of three replicates is shown. (**B**) On chip assay for visualizing eosinophil-derived EV incorporation by B16.F10 tumor cells by fluorescence microscopy. C16-labelled Eo5 or Eo33 and B16.F10 cells were loaded in the chip as indicated and incubated for 18 h. Images depict C16^+^ fluorescence (green) in B16.F10 cells at the beginning of the culture (T = 0 h) and after 18 h incubation. Inserts represent separate visible and green fluorescence channels. Scalebar: 100 μm. **C** Microphotographs showing incorporation of C16^+^ EV (green) from Eo5 or Eo33 in the cytoplasm of B16.F10 cells after 18 h. Chips were fixed and stained with DAPI (blue) at the end of the co-culture. Scalebar: 5 μm

Next, we assessed the incorporation of eosinophil-derived EV into a selection of tumor cells, namely B16.F10 melanoma cells, MCA205 sarcoma cells and TC1 lung adenocarcinoma cells. To this end, we co-cultured Bodipy FL C16-labeled Eo5 and Eo33 with tumor cells separated by a 0.4 μm pore-sized transwell system to allow the transfer of fluorescent EV without passage of cells (Fig. 2A). Flow cytometry analysis showed that fluorescent C16^+^ EV from Eo5 (Eo5-EV) and Eo33 (Eo33-EV) were incorporated rapidly (within 2 h) into B16 and MCA205 cells (> 95% C16^+^; Fig. 2A). In contrast, TC1 cells showed slower rate of EV incorporation, which was 50% for Eo5-EV and 60% for Eo33-EV at 2 h but reached > 95% C16 positivity after 18 h of culture (Fig. 2A). We further visualized the transfer of eosinophil-derived fluorescent EV into tumor cells by culturing Bodipy FL C16-labeled eosinophils and tumor cells in a microfluidic chip [21, 35]. In this setting, C16^+^ eosinophils and B16 tumor cells were embedded in Matrigel and loaded in separate lateral chambers connected by two arrays of microchannels and a central fluidic chamber to allow the passage of EV (Fig. 2B). After 18 h of co-culture in the chip, B16 acquired green fluorescence from either C16^+^ Eo5-EV or C16^+^ Eo33-EV to a similar extent, as shown by fluorescence microscopy (Fig. 2B). Time-lapse fluorescence microscopy was carried out to monitor the acquisition of green fluorescence by B16 tumor cells confirming efficient incorporation of either C16^+^ Eo5 or C16^+^ Eo33 by 5 h in this setting (Movies S1 and S2). Staining with DAPI at the end of the co-culture followed by confocal analysis and z-stack image acquisition, showed incorporation of fluorescent EV in the cytoplasm of B16 cells incubated with C16^+^ Eo5 or C16^+^ Eo33 (Fig. 2C; Figure S1; Movies S3 and S4). Overall, these findings demonstrate that both Eo5-EV and Eo33-EV are efficiently integrated into tumor cells.

### EV released by IL-33 activated eosinophils inhibit tumor proliferation by regulating cell cycle

We previously demonstrated that IL-33 promotes contact-dependent cytotoxic function of eosinophils against various cancer cell types [21, 22]. We asked whether the cytotoxic activity of IL-33 activated eosinophils could be mediated by EV. To this end, we co-cultured eosinophils with B16.F10 melanoma cells in the presence of GW4869 (GW), an inhibitor of EV biosynthesis [36], and analyzed apoptosis in tumor cells. Consistent with the different quantities of EV released by the two eosinophil populations, two μM GW were sufficient to inhibit EV secretion by Eo5 (66.4% inhibition; Figure S2), while efficient inhibition of EV release by Eo33 was achieved at doses of GW as high as 8 μM (69.5% inhibition; Figure S2). After 5 h, Eo33 induced the killing of significantly higher numbers of target tumor cells compared to Eo5 (Figure S3) confirming previous data [21, 22]. Pre-exposure of eosinophils to GW did not reduce the apoptosis rate induced by either Eo5 or Eo33 (Figure S3), ruling out an involvement of eosinophil-derived EV in eosinophil-mediated tumor cell killing.

**Fig. 3:**
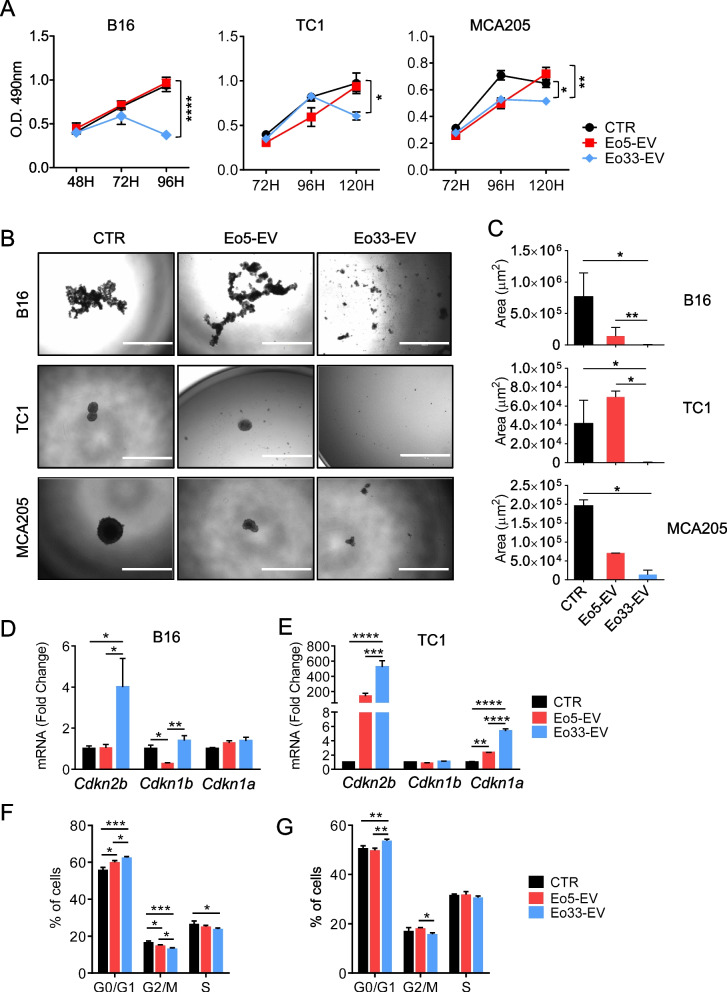
Anti-proliferative effects of EV from IL-33 activated eosinophils. **A** MTS assay of cell viability/proliferation in the indicated tumor cell lines cultured for various times alone (CTR) or in the presence of Eo5-derived EV (Eo5-EV) or Eo33-derived EV (Eo33-EV). Mean (SD) of five replicates is shown. *P < 0.05; **P < 0.01; ****P < 0.0001. **B-C** Tumor spheroid formation of indicated tumor cell lines cultured in ultralow attachment plates in medium alone (CTR) or with added Eo5-EV or Eo33-EV. **B** Representative microphotographs of tumor spheroids at day 4. Scalebar:1000 μm. **C** Quantitative analysis of tumor spheroid growth. Data show the mean spheroid area (SD) at the depicted time (*n* = 3–12). *P < 0.05; **P < 0.01. Expression of indicated CDKI protein-related genes in (**D**) B16 and (**E**) TC1 tumor cells cultured for 24 h alone (CTR) or in the presence of Eo5-EV or Eo33-EV. Mean (SD) of three replicates is shown. *P < 0.05; **P < 0.01; ***P < 0.001; ****P < 0.0001. Flow cytometry analysis of cell cycle in (**F**) B16 and (**G**) TC1 cells 24 h after exposure to Eo5-EV or Eo33-EV. Mean (SD) of three replicates is shown. *P < 0.05; **P < 0.01; ***P < 0.001

We sought to investigate the effects of eosinophil-derived EV on tumor cells. Exposure to Eo33-EV, but not to Eo5-EV, significantly inhibited the proliferation of B16, MCA205 and TC1 cells as revealed by MTS assay (Fig. 3A) and by reduction of tumor cell covered area in a crystal violet assay (Figure S4). Treatment of tumor cells with a 1:2 dilution of Eo33-EV (half) was sufficient to inhibit the proliferation of B16, TC1 and MCA205 cells (Figure S4), thus excluding the possibility that the anti-proliferative effects of Eo33-EV were ascribable to their major quantity. Moreover, in 3D culture models of B16, MCA and TC1 cells, exposure to Eo33-EV, but not Eo5-EV, markedly contrasted the formation of tumor spheroids (Fig. 3B and C). Since our data indicated that Eo33-derived EVs did not induce tumor apoptosis (Figure S3), we asked whether Eo33-EV inhibition of tumor cell proliferation could occur through blocking of cell cycle progression. Exposure to Eo33-EV, but not to Eo5-EV, up-regulated cyclin-dependent kinase inhibitor (CDKI) protein-related genes in tumor cells, specifically *Cdkn1b* and *Cdkn2b* in B16 cells (Fig. 3D) and *Cdkn2b* and *Cdkn1a* in TC1 cells (Fig. 3E). Furthermore, cell cycle analysis revealed significant increase of tumor cells in the G0/G1 phase and concomitant decrease of the proportion of cells in G2/M phase after Eo33-EV exposure compared with untreated and Eo5-EV treated B16 (Fig. 3F) and TC1 (Fig. 3G) tumor cells, indicating cell cycle arrest in G0/G1 phase. These data indicate that EV from IL-33 activated eosinophils inhibit tumor growth by blocking cell cycle.

### EV released by IL-33 activated eosinophils contrast malignant progression in target tumor cells

**Fig. 4:**
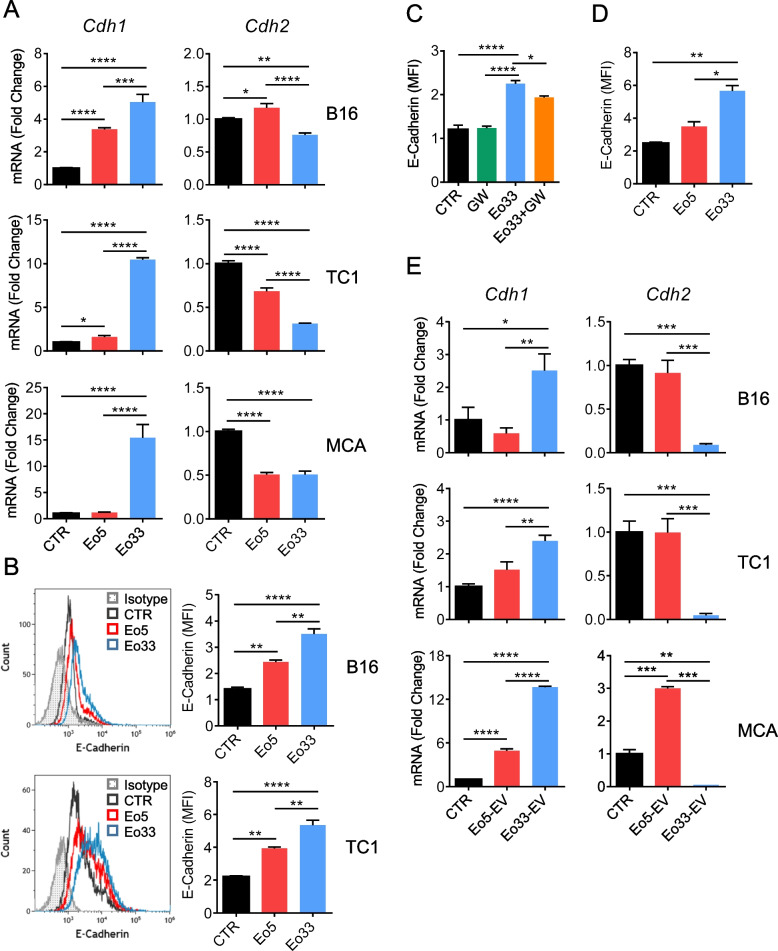
IL-33 activated eosinophils regulate EMT markers through EV. Tumor cells were cultured for 24 h alone (CTR) or in the presence of Eo5 or Eo33 (4:1 Eo:Tum ratio). **A** Expression of *CDH1* (E-Cadherin) and *CDH2* (N-Cadherin) genes in the indicated cell lines. Mean (SD) of three replicates is shown. *P < 0.05; **P < 0.01; ***P < 0.001; ****P < 0.0001. **B** Flow cytometry analysis of the expression levels of E-Cadherin on tumor cells. Left, representative histograms. Right, mean fluorescence intensity (MFI) of three replicates, mean (SD). **P < 0.01; ****P < 0.0001. E-Cadherin expression on B16 melanoma cells (**C**) following 24 h co-culture with GW4869 pre-treated or untreated Eo33 or (**D**) cultured with Eo33 in a 0.4 μm Transwell system (24 h). Mean (SD) of three replicates is shown. *P < 0.05; **P < 0.01; ****P < 0.0001. **E** Expression of *CDH1* and *CDH2* in the indicated tumor cell lines cultured for 24 h alone (CTR) or in the presence of Eo5-EV or Eo33-EV. Mean (SD) of three replicates is shown. *P < 0.05; **P < 0.01; ***P < 0.001; ****P < 0.0001

**Fig. 5:**
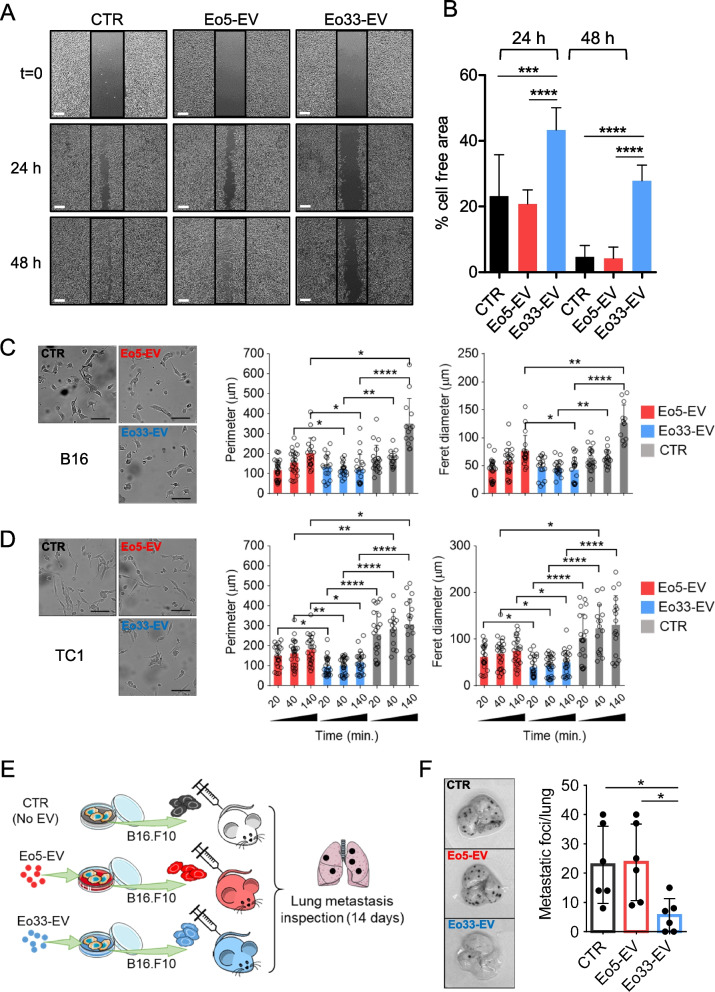
Phenotypic changes in tumor cells following incorporation of Eo33-EV. **A** Wound-healing scratch assay. B16 melanoma cells were exposed to Eo5-EV, Eo33-EV or left untreated for 24 h and then scratched. Phase-contrast pictures were taken at the indicated times. Scalebar, 100 μm. **B** Quantification of cell-free area by ImageJ analysis at indicated times. Mean (SD) of several fields is shown. ***P<0.001; ****P < 0.0001. Morphologic changes in (**C**) B16 melanoma cells and (**D**) TC1 lung adenocarcinoma cells after exposure to Eo5-EV or Eo33-EV. Representative microphotographs at 140 min of culture (left); scalebar, 100 μm. Feret’s diameter and cell perimeter (right) were extrapolated from time lapse video (1440 min., frame interval 20 min). Histograms represent the mean values (SD) of several cells from several fields. Dots represent single cells. *P < 0.05; **P < 0.01; ***P < 0.001; ****P < 0.0001. **E **Experimental melanoma pulmonary metastasis assay. B16 melanoma cells were exposed to Eo5-EV, Eo33-EV or left untreated for 24 h and then injected intravenously (2 × 10^5^) in C57Bl/6 mice. Mice were sacrificed 14 days later to examine metastases formation in the lungs. **F** Representative images of metastatic lungs for each experimental group (left) and lung metastatic foci counts (right). Mean (SD) is shown (*n* = 6 mice/group). *P < 0.05

Eosinophil-derived EV have been shown to guide airway remodeling in asthma by altering lung epithelial cell gene expression [31]. The ability of eosinophil-derived EV to affect the transcriptional program of target cells prompted us to investigate the effects of Eo33-EV on tumor cell phenotype. Co-culture of mouse tumor cells with Eo33, compared to Eo5, induced more substantial up-regulation of the epithelial marker E-Cadherin/*Cdh1* and down-modulation of the mesenchymal molecule N-Cadherin/*Cdh2* (Fig. 4A). Flow cytometry analysis confirmed higher expression of E-Cadherin on the tumor cell membrane following co-culture with Eo33, with respect to Eo5 co-cultured or control tumor cells (Fig. 4B). Of note, Eo33-mediated up-regulation of membrane E-Cadherin was partially inhibited by GW (Fig. 4C) and was observed also in absence of direct contact between tumor cells and eosinophils (Fig. 4D), indicating that the modulatory effect could be mediated by EV. Consistently, exposure to Eo33-EV also induced up-regulation of *Cdh1* and down-regulation of *Cdh2* in tumor cells (Fig. 4E). To evaluate whether the transcriptional reprogramming following Eo33-EV incorporation resulted in a less invasive phenotype of tumor cells we conducted a scratch assay. As shown in Fig. 5A, B16 melanoma cells untreated or pre-exposed to Eo5-EV almost replenished the wound caused by the scratch as soon as after 24 h of culture. In contrast, tumor cells pre-exposed to Eo33-EV showed significant delay in closure rate up to 48 h (Fig. 5A-B). Of note, pre-exposure of tumor cells to Eo33-EV was sufficient to inhibit melanoma migration in the wounded area, since a second administration of Eo33-EV during the scratch assay did not enhance this effect further (Figure S5). We analyzed the effects of eosinophil-derived EV in shaping tumor cell morphology as possible predictor parameter for metastatic state [37]. Morphometric analysis of tumor cells showed increased elongation (Feret diameter) and perimeter in B16 (Fig. 5C) and TC1 cells (Fig. 5D) after 140 min of culture alone or in the presence of Eo5-EV, indicating changes towards an invasive phenotype. In contrast, B16 (Fig. 5C) and TC1 (Fig. 5D) tumor cells receiving Eo33-EV maintained their morphometric parameters virtually unchanged over this time. We further evaluated whether the phenotype acquired by tumor cells following Eo33-EV reprogramming affected their metastatic potential in vivo in a melanoma experimental pulmonary metastasis model. To this end, we intravenously injected B16.F10 melanoma cells, either untreated or treated with Eo33-EV or Eo5-EV, into syngeneic C57Bl/6 mice and analyzed metastatic colonization in lungs 14 days later (Fig. 5E). Remarkably, mice injected with Eo33-EV treated B16.F10 melanoma cells developed a significantly reduced number of pulmonary metastases with respect to mice injected with Eo5-EV reprogrammed or control cells (Fig. 5F). Overall, these results indicate that EV from IL-33 activated eosinophils affect epithelial to mesenchymal transition (EMT)-related gene program in target tumor cells shaping their phenotype towards a less metastatic potential.

### Comparative analysis of Eo5-EV and Eo33-EV molecular content by RNA-Seq

**Fig. 6:**
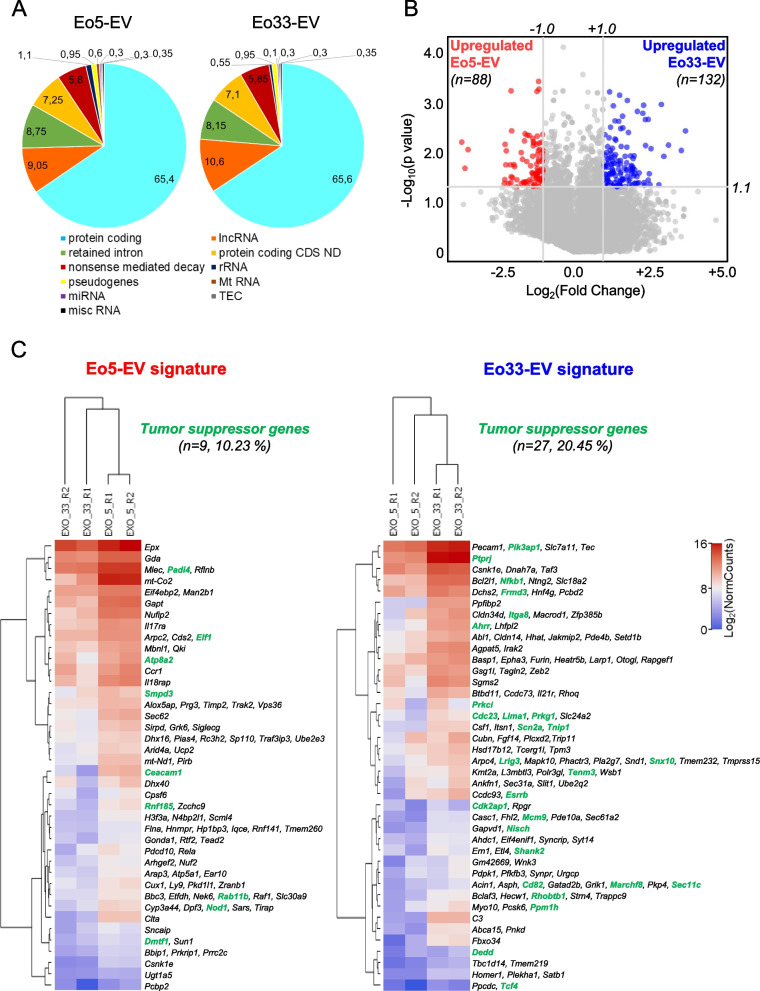
RNA-Seq analysis of Eo-EV signatures. **A** RNA Transcript biotypes distribution into the two indicated EV signatures. Classification was performed according to the ENSEMBL official transcript definition (https://www.ensembl.org/info/genome/genebuild/biotypes.html). Values depict the percentages of the indicated biotypes, expressed as the mean read per each biotype versus the total number of reads. **B** Distribution of differentially expressed protein-coding mRNAs alongside the indicated Eo-EV signatures. Volcano plot shows the *p* values (Y axis, negative base 10 logarithm) distribution versus the mean Fold Change (X axis, base 2 logarithm). Colored dots represent significant (-log10(*p* value) > 1.3) mRNAs whose mean FC is upregulated (log2(Fold Change) > 1, Eo33-EV) or downregulated (log2(Fold Change) < 1, Eo5-EV). Grey lines represent the *p* value and Fold Change settings employed to select statistically significant upregulated/downregulated mRNAs (italic numbers). **C** Differential expression of the significant genes (as delineated in panel B) for the Eo-EV signatures compared to the two experimental conditions. Heatmap shows the base 2 logarithm of the 88 and 132 differentially expressed genes into the replicates (R1, R2) for each Eo-EV signature. Dendrograms show hierarchical clustering for experimental conditions (EXO5 for Eo5-EV, EXO33 for Eo33-EV) and mRNAs. Gene expression has been clustered by K-means method (*n* = 60). Tumor suppressor genes (provided by the CancerMine and TSGene databases) are indicated for each Eo-EV signature with their respective percentage (number of tumor suppressors versus total genes into the signature)

To discover the molecular content enclosed within eosinophil-derived EV accounting for the observed effects on cancer cells, we carried out bulk RNA-Seq on Eo5-EV and Eo33-EV and on their respective producing cells (Eo5 and Eo33). RNA-Seq identified 149,222 transcripts in our samples, which take into account for ~65% protein coding transcripts (mRNA) and for ~10% long non-coding RNAs (lncRNA) of Eo-EV raw read count, whereas only a small percentage (0.3%) of microRNAs was found (Fig. 6A). By multiple cross-filtering process [log_2_ (normalized counts) > 0], we obtained lncRNA and mRNA transcripts expressed in EV (either Eo5-EV or Eo33-EV) and simultaneously in their producing cells (Eo5 or Eo33, respectively). Among the lncRNA, we found 11 transcripts up-regulated in Eo5-EV corresponding to 11 Ensembl gene IDs and 23 lncRNA in Eo33-EV corresponding to 23 Ensembl gene IDs, albeit all of unknown function (Figure S6). Through the same procedure, we identified 88 mRNAs significantly up-regulated in Eo5-EV and 132 mRNAs significantly up-regulated in Eo33-EV whose expression was detected also in the producing cells, Eo5 and Eo33 (Fig. 6B; Figure S7). Hierarchical clustering analysis on heatmap (Fig. 6C) underscored a close similarity between the two experimental replicates (R1, R2) within each condition (Eo5-EV or Eo33-EV). Moreover, combined CancerMine plus TSGgene data mining underscored a significantly (*P* = 0.0244) higher number of tumor suppressor genes in the Eo33-EV signature (*n* = 27) accounting for 20.45% of total mRNA transcripts (Fig. 6C) with respect to those detected in the Eo5-EV signature (*n* = 9; 10.23%; Fig. 6C).

Based on the selected gene sets, we found Eo33-EV transcripts enriched in Gene Ontology (GO) pathways linked to general homeostatic cell functions, such as import into cell (GO:0098657), establishment of localization (GO:0051234), vesicle-mediated transport (GO:0016192), and intracellular signal transduction (GO:0035556), as summarized in Table 1 (cyan). We also found enrichment in negative regulation of cellular processes (GO:0048523) compatible with the inhibition or delay of cell proliferation and a general decrease of cell activity (Table 1, cyan). Moreover, Eo33-EV transcripts were enriched in pathways associated to epithelial phenotypes, such as cell junction organization (GO:0034330) and actin filament-based process (GO:0030029) (Table 1, cyan). In contrast, Eo5-EV transcripts were enriched in pathways associated to cell cycle activation (Table 1, red), such as positive regulation of cellular processes (GO:0048522), positive regulation of biological processes (GO:0048518) and positive regulation of metabolic processes (GO:0009893). Other GO terms found enriched in Eo5 transcripts included innate immune response (GO:0045087) regulation of gene expression (GO:0010468) and cellular response to chemical stimulus (GO:0070887).

**Table 1:**
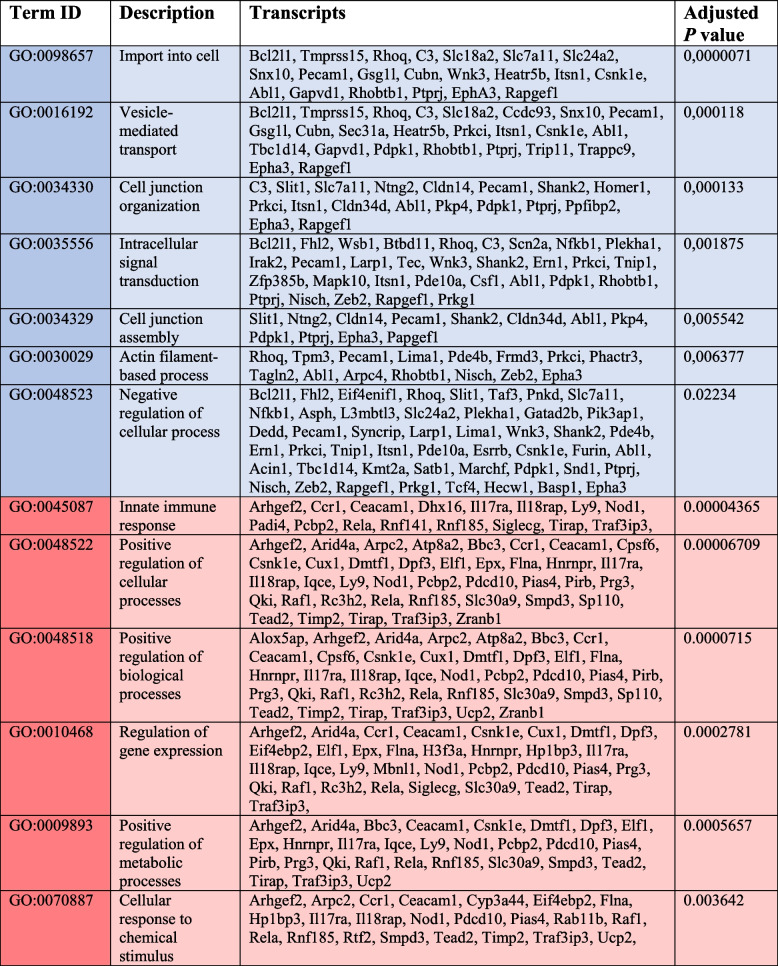
GO pathway analysis of mRNAs enriched in Eo33-EV vs Eo5-EV

Cyan: pathways enriched in Eo33-EV; Red: pathways enriched in Eo5-EV

### EV from IL-33 activated human eosinophils reprogram human melanoma cells towards a less aggressive phenotype

**Fig. 7:**
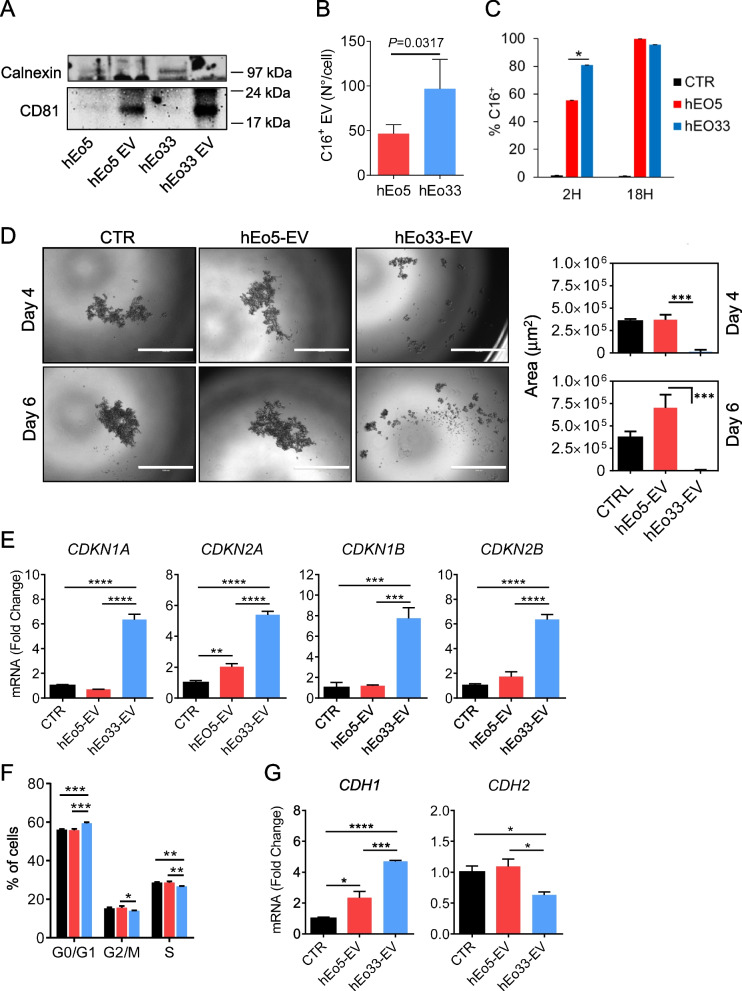
IL-33 stimulates EV secretion by human eosinophils that reprogram human melanoma cells. **A** Western blot analysis of CD81 and Calnexin expression in human eosinophils stimulated with IL-5 (hEo5) or IL-33 (hEo33) and their derived EV (hEo5-EV and hEo33-EV). **B** Flow cytometry quantification of fluorescent EV generated by Bodipy FL-C16 labelling of hEo5 and hEo33. Data are expressed as number of EV released per cell. Mean (SD) of three experiments is shown. **C** Incorporation of fluorescent eosinophil-derived EV into A375P melanoma cells following co-culture with C16-labelled hEo5 or hEo33 in 0.4 Transwell system for the indicated times. Mean (SD) of three replicates is shown. **D** Tumor spheroid formation of A375P human melanoma cells cultured alone (CTR), with hEo5-EV or hEo33-EV. Left, representative micrographs at the indicated times. Bars: 1000 μm. Right, quantitative analysis of tumor spheroid area. Mean (SD) of several spheroids is shown. ***P < 0.001. **E** Gene expression analysis of CDKI in A375P cells following 24 h exposure to hEo5-EV or hEo33-EV. Mean (SD) of three replicates is shown. **P < 0.01; ***P<0.001; ****P < 0.0001. **E** Cell cycle analysis in A375P cells after 24 h exposure to hEo5-EV or hEo33-EV. Mean (SD) of three replicates is shown. **P < 0.01; ***P < 0.001. (**F**) Expression of *CDH1* and *CDH2* genes in A375P cells exposed to hEo5-EV or hEo33-EV (24 h). Mean (SD) of three replicates is shown. *P < 0.05; ***P < 0.001. ****P < 0.0001

In order to translate the findings with the murine model into the human system, we carried out studies with human eosinophils and human tumor cells. Eosinophils purified from the peripheral blood of healthy donors were stimulated for 18 h with IL-5 (hEo5) or IL-33 (hEo33). Co-culture of hEo33 with A375P melanoma cells markedly reduced tumor cell growth compared to cells cultured in the presence of hEo5 (Figure S8A and S8B). Following co-culture with hEo33, A375P cells exhibited increased levels of *CDH1* while down-regulated *CDH2*, with respect to melanoma cells co-cultured with hEo5 or control (Figure S8C). Furthermore, co-culture with hEo33, but not with hEo5, increased the expression of *CDKN1A* and *CDKN1B* in A375P cells (Figure S8D). These observations indicate that also for human cells IL-33 activation of eosinophils produces phenotypic alterations in adjacent tumor cells. To evaluate the role of eosinophil-derived EV in these phenomena, we isolated and characterized EV from hEo5 (hEo5-EV) and hEo33 (hEo33-EV) culture media. The purity of hEo5-EV and hEo33-EV was confirmed by expression of the EV marker CD81 and lack of Calnexin, whose levels were found only in the producing eosinophils (Fig. 7A). Notably, the expression of CD81 was higher in hEo33-EV with respect to hEo5-EV (Fig. 7A). We enumerated hEo5-EV and hEo33-EV by Bodipy FL C16 labeling of human eosinophils. Flow cytometry analysis revealed that hEo33 released higher numbers of C16^+^ EV with respect to hEo5 (twofold; Fig. 7B). We analyzed the transfer of fluorescent eosinophil-derived EV into A375P melanoma cells and found, after 2 h, increased incorporation of C16^+^ hEo33-EV (80%; Fig. 7C) with respect to C16^+^ hEo5-EV (60%; Fig. 7C), both reaching 100% incorporation by 18 h (Fig. 7C). We next evaluated the ability of human eosinophil-derived EV to shape the phenotype of A375P cells. We found that exposure to hEo33-EV, but not to hEo5-EV, markedly contrasted the formation of A375P tumor 3D spheroids over time (Fig. 7D) and effectively blocked tumor proliferation even when diluted 1:2 (Figure S9). Consistently, hEo33-EV, but not to hEo5-EV, induced in A375P cells up-regulation of the CDKI genes *CDKN1A*, *CDKN2A*, *CDKN1B* and *CDKN2B* (Fig. 7E) and block of cell cycle in G0/G1 phase (Fig. 7F). Moreover, A375P melanoma cells significantly increased the expression of *CDH1* with concomitant down-modulation of *CDH2* in the presence of hEo33-EV, with respect to cells exposed to hEo5-EV or left untreated (Fig. 7G). Taken together, these data indicate that similarly to mouse eosinophils, activation of human eosinophils with IL-33 stimulates the secretion of EV that induce transcriptional reprogramming in receiving tumor cells towards a block of cell proliferation and of EMT-related markers.

## Discussion

Despite the controversial role of IL-33 in cancer immunity, it is extensively demonstrated that this epithelial-derived alarmin may provide anticancer responses in a number of tumors due to its ability to stimulate a wide set of immune cells, such as CD8^+^ T cells, NK, DCs, eosinophils and basophils that infiltrate the TME [38]. Eosinophils have emerged as important components of the TME exerting several functions against cancer, ranging from the secretion of soluble mediators (i.e., chemokines) that facilitate the recruitment of CD8 + T cells at the tumor site to cytotoxic degranulation [19, 21, 22, 24, 39]. On the other hand, eosinophils can secrete several pro-angiogenic factors, such as VEGF-A, fibroblast growth factor 2 (FGF-2) and CXCL8/IL-8, which may favor tumor progression [19, 40]. In this scenario, EV as conveyors of molecular messages may intervene providing communication between tumor and immune cells, including eosinophils, affecting tumor fate [5]. In the present study, we provide the first evidence of a role for eosinophil-derived EV in controlling tumor proliferation and metastatic potential through transcriptional reprogramming of target tumor cells.

First, we demonstrate that stimulation with IL-33 increased the secretion of EV by both mouse and human eosinophils, as shown by flow cytometry-based enumeration of fluorescent Bodipy FL C16^+^ EV [33, 34] and by increased levels of EV associated proteins in Eo33-EV with respect to Eo5-EV. It has been described that under inflammatory conditions such as asthma, eosinophils release EV, which participate in asthmatic airway remodeling by affecting inflammation-related gene expression in target cells [29, 30]. Moreover, EV secretion by human eosinophils is enhanced following activation with IFN-γ [29], CCL11 or TNF-α [41]. These observations suggest that activation stimuli promote EV secretion by eosinophils, consistent with the notion that the quantity of secreted EV depends on the functional status of the producing cell [1].

Once incorporated into recipient cells, EV can activate downstream signaling cascades that result in transcriptional reprogramming and modifications of cellular physiology [3, 4]. We found that mouse and human IL-33 activated eosinophil-derived EV affect the transcriptional program of target tumor cells towards a block of cell cycle and malignant progression. Tumor progression represents a dense network of multiphasic proceedings that may result in the constitutive activation of genes involved in uncontrolled cell proliferation and redundant cell cycle events. We demonstrate that EV from Eo33 drive the expression of genes coding for CDKI proteins in mouse and human tumor cells that prevent cell cycle progression from G1 to S phase and act as tumor suppressor genes [42]. This transcriptional reprogramming corresponded to cell cycle block in G0/G1 phase and an effective decrease in the proliferative rate of cancer cells, revealed by MTS and crystal violet assays and by inhibition of tumor spheroid formation in 3D culture system. Of note, the anti-proliferative effects of Eo33-EV did not seem to involve tumor apoptosis, since blockade of EV generation by GW4869 did not prevent eosinophil-induced tumor cell death. This result is consistent with previous reports by our group [21] and others [26, 43] demonstrating that the cytotoxic activity of eosinophils is both contact-dependent and relies on degranulation and release of lytic proteins (i.e., ECP, EPX and granzyme B).

The analysis of the main molecules that regulate EMT, namely E-Cadherin and N-Cadherin [44] denoted a reversion of this process in mouse and human tumor cells following exposure to IL-33 activated eosinophil-derived EV. In fact, A375P melanoma cells up-regulated the expression of *CDH1* while down-modulated *CDH2* after exposure to hEo33-EV or co-culture with hEo33. Similarly, mouse tumor cells co-cultured with Eo33 or exposed to Eo33-EV expressed significantly higher levels of *Cdh1* gene and surface E-Cadherin and with concomitant down-modulation of *Cdh2*. Loss of E-cadherin expression and acquisition of N-cadherin are critical steps in cancer dissemination [44]. E-cadherin, as a key component of adherens junctions, plays a major role in maintaining adherence between adjacent cells [45]. Transcriptional “switch” from E-Cadherin to N-cadherin expression in cancer cells results in acquisition of a mesenchymal phenotype characterized by loss of cell–cell junctions and acquisition of elongated shape, increased motility and invasive capacity [44, 46]. The transcriptional reprogramming of tumor cells following Eo33-EV incorporation correlated with a reduced migration/motility in a scratch assay and the failure to acquire an elongated shape in vitro and, of more relevance, with a lower metastatic propensity in vivo. The requirement of EMT for the formation of cancer metastases has been long debated [46, 47]. Previous studies showed that inactivation of EMT inducer-transcription factors promote metastatic colonization to distant sites [48–50]. On the other hand, recent reports demonstrated a positive association between EMT-related markers with increased potential for metastases formation in mouse models of melanoma [51–53], squamous cell carcinoma [54] and lung cancer [55, 56]. These contrasting observations may be attributed to the transient nature of EMT process, which may be reversed upon reception of specific signals at the metastatic site.

Although IL-33 activated eosinophils released higher quantities of EV, our on chip and transwell experiments with Bodipy FL C16-labelled mouse eosinophils indicate that both Eo5-EV and Eo33-EV were efficiently integrated into target tumor cells. These observations indicate that the transcriptional reprogramming of tumor cells induced by Eo33-EV was not attributable to increased quantities of EV incorporated but rather by the molecular cargo composition, as also indicated by the RNA-Seq analysis. We uncovered distinct transcript profiles in Eo5-EV and Eo33-EV that were compatible with the observed effects on tumor cell phenotype shaping. In fact, an increased number and percentage of tumor suppressor genes were found in the Eo33-EV signature with respect to Eo5-EV transcripts. Among mRNAs preferentially expressed in Eo33-EV, we retrieved a number of transcripts whose functions could counteract tumor transformation and progression. The metastasis suppressor *cd82* is known to reverse EMT process in cancer cells by inhibiting the TGF-β1/Smad andWnt/β-catenin pathways [57]. The tumor suppressor gene *Nisch* drives transcriptional up-regulation of E-cadherin and, contextually, downregulation of several mesenchymal genes, including N-cadherin in cancer cells [58] and was shown to be delivered through EV conferring traits of reduced migration, adhesion, and spreading in target cancer cells [59]. Some of the mRNAs preferentially expressed in Eo33-EV are involved in negative regulation of cellular activities. In particular, *Acin1*, *Ahrr*, *Basp1*, *Bclaf3*, *Dedd, Esrrb, Fhl2, Mapk10, Satb1, Taf3* can negatively regulate the transcriptional machinery of cells by acting at different levels, such as through a negative regulation of transcription by RNA polymerase II [60–62] or through a negative regulation of DNA-templated transcription [63, 64].

The mRNA signature of Eo33-EV corresponded to enrichment in GO biological processes such as actin filament-based process, cell junction organization and cell junction assembly that involve tight junction and adherens junction, the latter primarily composed of E-cadherin [45]. Defects in cell–cell junctions give rise to a wide range of tissue abnormalities and are common in cancers. This trait correlates with the observed activity of Eo33-EV in inducing an epithelial-like phenotype in tumor cells by affecting the expression of E-Cadherin and N-Cadherin. Through GO analysis, we also found Eo33-EV transcripts associated with “negative regulation of signaling” that may lead to a possible decrease of tumor cell activities, such as metabolism and proliferation. Among the mRNAs significantly more up-regulated in Eo33-EV, we also found transcripts involved in cell cycle regulation. These include *Cdk2ap1,* which negatively regulate CDK2 activity resulting in cell cycle arrest in the G1 phase [65]. The F-box protein FBXO34 is a substrate-recognition component of the SCF (SKP1-CUL1-F-box protein)-type E3 ubiquitin ligase complex involved in cell cycle regulation [66] that has been reported to regulate both the G2/M transition and anaphase entry [67]. The protein tyrosine phosphatase receptor type J (PTPRJ) exerts a negative regulatory effect on cell proliferation, migration, differentiation, and cell adhesion, and is therefore considered a tumor suppressor [68]. In contrast, Eo5-EV signature showed enrichment in pathways generally associated to cell activation, cell proliferation and metabolic functions. In particular, we found the presence of transcripts, such as *Cux1* and *Arpc2*, which variably regulate DNA replication, cell proliferation and motility [69, 70] and *Csnk1e,* which is implicated in the Wnt/β-catenin pathway, a key determinant in cancer proliferation [71].

Besides mRNAs, other molecular determinants present in the Eo33-EV cargo may account for the transcriptional reprogramming of tumor cells. Several studies have demonstrated the effects of various immune cell-derived EV on tumor cells through specific miRNAs, lncRNAs or proteins contained in the EV cargo [5]. Our RNA-seq analysis returned only a small portion of miRNA (0.3%) in the eosinophil-derived EV cargo, with no significant miRNA differentially expressed in the two Eo5-EV and Eo33-EV populations. With regard to lncRNA, we could not find documented function for any of the transcripts up-regulated in either Eo5-EV or Eo33-EV. Therefore, it is possible that lncRNAs and/or proteins, which were not analyzed in this study, also contribute to the Eo33-EV induced effects on tumor cells.

Of relevance, we could translate to the human system the findings that EV from IL-33-activated human eosinophils induced transcriptional reprogramming of cell cycle and EMT-related genes resulting in anti-proliferative effects on A375P melanoma cells in standard and 3D cultures. We observed a more rapid incorporation of hEo33-EV at 2 h, with respect to hEo5-EV. However, both hEo5-EV and hEo33-EV were efficiently incorporated by 18 h. Given that our analyses on gene expression and tumor phenotype changes were performed at times > 24 h, it is unlikely that the differential effects of hEo33-EV as opposed to hEo5-EV could be attributable to different amounts of incorporated vesicles by the tumor cells. Moreover, the observation that half of hEo33-EV adjusted to the same amount as hEo5-EV were as efficient as undiluted hEo33-EV in blocking the proliferation of A375 cells (as observed also in the mouse system) supports the concept that the differential effects of hEo33-EV and hEo5-EV are qualitative rather than quantitative. In this respect, a molecular profiling of hEo33-EV and hEo5-EV cargos would clarify the homology of the determinants with the murine system.

Our findings provide novel mechanistic insights on the function of eosinophils in the tumor microenvironment that may underlie the diverse prognostic role of these cells in human cancers [19]. Recent evidences suggest that the TME comprise a heterogeneous population of eosinophils of different origin (i.e., resident *vs* inflammatory) and with plastic functions in response to environmental factors that may induce different transcriptional programs in these cells [24, 72]. It has been reported that activation of eosinophils with certain stimuli, such as IFN-γ, associates both with enhanced anti-tumor functions [24] and with increased EV secretion [29]. In this context, our study provides the first demonstration of a link between anti-tumor activities of eosinophils mediated by EV release following activation with IL-33. The intrinsic properties of EV in regulating intracellular pathways have advanced their potential utility in the therapeutic control of many diseases and particularly in cancer. The future of EV-based therapy includes engineered immune-derived EV and combinations of targeted EV with anticancer drugs for in vivo tracking, prognosis monitoring and cancer therapy [73]. In this respect, the GO enrichment in Eo33-EV transcripts in pathways involving import into cell (GO:0098657) and vesicle-mediated transport processes (GO:0016192) may suggest a greater potential of Eo33-EV for drug transport. In conclusion, our study provides a possible mechanism by which IL-33 stimulates the anticancer activities of eosinophils through EV-mediated reprogramming of tumor cells and opens new perspectives on the use of eosinophil-derived EV in cancer therapy.

## Materials and methods

### Tumor cell lines

Murine B16.F10 metastatic melanoma cells (ATCC, CRL-6475), TC1 lung adenocarcinoma cells (provided by Dr. Guido Kroemer, Gustave Roussy Cancer Institute, France), MCA205 fibrosarcoma cells (Merk Millipore, SCC173) and human metastatic melanoma A375P cells (ATCC, CRL-3224) were used in this study. B16.F10 and A375P were cultured in DMEM High Glucose supplemented with 10% FBS, 1% glutamine, 1% Antibiotic–Antimycotic (all from Euroclone). MCA205 were maintained in RPMI 1640 (EuroClone) supplemented with 10% FBS, 1% glutamine, 1% Antibiotic–Antimycotic. TC1 were cultured in RPMI 1640 supplemented with 10% FBS, 1% glutamine, 1% Antibiotic–Antimycotic plus 1 mM sodium pyruvate, 10 mM Hepes and 1X non-essential aminoacids (NEAA). All cell lines were constantly tested for morphology, absence of mycoplasma and passaged for no more than 3–4 times from thawing.

### Generation of bone marrow-derived eosinophils

Bone marrow (BM) -derived murine eosinophils were obtained following a differentiation protocol already described [21] starting from BM cells from naïve C57Bl/6 mice. BM cells were extracted from both tibia and femur and treated with ACK lysis buffer (140 mM NH_4_Cl, 17 mM Tris HCl, pH 7.2) to discard erytrocites. Cells were plated at 2 × 10^6^/mL in RPMI 1640 containing 20% FBS, 1% glutamine, 1% Antibiotic–Antimycotic, 10 mM Hepes, 1X NEAA, 1 mM sodium pyruvate, supplemented with 100 ng/mL rmSCF (Cell Guidance Systems) and 100 ng/mL rmFLT3-L (Cell Guidance Systems) and cultured at 37 °C in 5% CO_2_. On day 4, 10 ng/mL rmIL-5 (PeproTech) were added directly to the culture media. On day 8, cells were harvested, counted and splitted (2 × 10^6^/mL) in two new T75 flasks containing fresh medium supplemented with 10 ng/mL rmIL-5. At days 10 and 12, 5 mL of fresh medium containing 10 ng/mL rmIL-5 were added. On day 14, cells were harvested, counted and replated 2 × 10^6^/mL in a new flask containing fresh medium and supplemented with 10 ng/ml rmIL-5 to generate a population of IL-5 eosinophils (Eo5) or with 100 ng/ml rmIL-33 (Biolegend) to generate a population of IL-33 eosinophils (Eo33). Cells are used on day 15 or 16, after overnight incubation with 10 ng/ml rmGM-CSF (Miltenyi Biotec), directly added in the culture media. At the end of differentiation, eosinophil purity (> 80%) was tested as already described [21].

### Isolation and stimulation of human eosinophils

The Ethics Committee of the Istituto Superiore di Sanità (Rome, Italy) approved the study protocol involving the use of human blood cells (Prot. n. OO-ISS 02/10/2019 0029604). Buffy coats from healthy donors (hepatitis B surface antigen, hepatitis C virus and HIV virus negative) were used to isolate human eosinophils. Leukocytes were separated from erythrocytes by dextran sedimentation (Dextran 70 BioChemica A1847, PanReac Applichem). Granulocytes were obtained by density gradient centrifugation of leukocytes in Lymphosep, Lymphocyte Separation Media (Aurogene). Eosinophils were then isolated by negative immunomagnetic selection by using Human Eosinophil Isolation Kit (Miltenyi Biotec). Eosinophils were seeded 2 × 10^6^/mL in RPMI 1640 containing 1% glutamine, 1% Antibiotic–Antimycotic, 20% FBS, 25 mM Hepes, 1X NEAA, 1 mM sodium pyruvate in the presence of either 10 ng/mL rhIL-5 (Cell Guidance Systems) or with 50 ng/mL rhIL-33 (Prospec-Tany Technogene Ltd.) and incubated at 37°C in 5% CO_2_ for 24 h.

### Isolation of EV from mouse and human eosinophils

For isolation of EV from mouse eosinophils, conditioned medium of Eo5 and of Eo33 at day 16 were subjected to centrifugations (1400 rpm 10 min at 4 °C, then 3500 rpm 20 min 4 °C) to remove intact cells and debris. Subsequently, conditioned media were subjected to serial ultracentrifugations in a SW41 Ti swinging bucket rotor (BeckmanCoulter): 10,000 × g (20 min at 4 °C) to remove granules and larger EV followed by 100,000 × g (2 h at 4 °C) to collect the resulting pellet of Eo5- and Eo33-derived small EV (Eo5-EV and Eo33-EV, respectively) [33]. The EV pellets were washed with PBS 100,000 × g 2 h at 4 °C before use.

For isolation of EV from human eosinophils, conditioned media from human eosinophils stimulated with IL-5 (hEo5) or with IL-33 (hEo33) were harvested and centrifuged 1400 rpm for 10 min at 4 °C to remove cells and debris. Intact EV with a size ranging from 40–200 nm were purified by employing the Cell Culture Media Exosome Purification Kit (Norgen Bioteck Corp.). EV were then resuspended in PBS or fresh medium depending on specific experimental requirements.

### Generation and quantification of Bodipy FL C16 labeled fluorescent EV

We generated fluorescent EV by labeling eosinophils with Bodipy FL C16 (4,4-difluoro5,7-dimethyl-4-bora-3a,4a-diaza-s-indacene-3-hexadecanoic acid, C16; Thermo Fisher Scientific), a fatty acid analogue that enters the cellular lipid metabolic pathway without affecting the natural lipid metabolism and homeostasis inside the cell. Bodipy FL C16 rapidly integrates into major phospholipid classes producing green fluorescent EV as a direct result of biogenesis [33, 34]. Mouse and human Eo5 and Eo33 were seeded 1 × 10^6^/mL in a 0,3% FBS RPMI 1640 cell-labeling medium containing 7 μM Bodipy FL C16 and were incubated for 4 h at 37 °C in 5% CO_2_. For human eosinophils, medium was harvested and stored at 4 °C. For murine eosinophils, cells were washed and seeded at 1 × 10^6^ cell/mL in complete RPMI medium in the presence or absence of 0.05 × 10^6^/mL B16.F10 melanoma cells were added to the culture (Eo:Tumor cells ratio 20:1). Following 24 h incubation, C16^+^ EV-containing conditioned medium were harvested and ultracentrifuged at 10,000 × g, 4 °C to remove granules and larger EV. Aliquots of appropriately diluted fluorescent mouse and human C16^+^ Eo5 or C16^+^ Eo33 EV-containing conditioned medium were analyzed and enumerated through flow cytometry, by employing CytoFlex (Beckman Coulter) and analyzed with the CytExpert Analysis Software (Beckman Coulter). Inhibition of EV secretion was evaluated by treatment of eosinophils with GW4869 (N,N’-Bis[4-(4,5-dihydro-1H-imidazol-2-yl)phenyl]-3,3’-p-phenylene-bis-acrylamide dihydrochloride) (Sigma), an inhibitor of extracellular vesicle biogenesis and release [36]. Eosinophils were incubated for 4 h with 7 μM Bodipy FL C16 in labelling medium containing or not increasing concentrations of GW4869. Cells were washed and further incubated with GW4869 for 4 h before conditioned medium collection. C16^+^ EV were isolated and enumerated by flow cytometry.

### Uptake of eosinophil-derived EV by target tumor cells

The incorporation of secreted C16^+^ Eo5-EV and C16^+^ Eo33-EV within tumor cells was evaluated using 0.4 µm pore-sized cell culture inserts (Corning Costar Corporation) that allows the transit of EV but not of cells. Mouse and human Eo5 and Eo33 were labeled with C16, as described above. Mouse tumor cells (B16.F10, TC1, MCA205) were seeded (2 × 10^5^ in 600 µL) in the bottom compartment of the 24-well plate while C16^+^ Eo5 and C16^+^ Eo33 were plated (2 × 10^6^ cells in 100 µl) in the upper insert (Eo:Tumor cells ratio, 10:1). For human experiments, A375P cells were seeded (1 × 10^5^ in 600 µl) in the bottom compartment whereas C16^+^ Eo5 and C16^+^ Eo33 (1 × 10^6^ in 100 µl) were placed in the upper transwell insert (Eo:Tumor cells ratio, 10:1). Controls consisted of tumor cells cultured in the absence of eosinophils. The incorporation of secreted C16^+^ Eo5-EV and C16^+^ Eo33-EV into tumor cells was evaluated at various times of culture by fluorescence microscopy and/or flow cytometry through analysis of acquisition of green fluorescence by tumor cells.

Microfluidic devices were used to evaluate EV incorporation in time lapse. Briefly, ad hoc fabricated chips [21, 35] composed of a central chamber connected to two minor side chambers by a set of micro-size channels were loaded with B16.F10 cells (2 × 10^4^ in 5 ul Matrigel 2,5 mg/ml) and C16^+^Eo5 or C16^+^Eo33 (1 × 10^5^ cells) as depicted in Fig. 2B. Lateral chambers were filled with medium to stabilize the microfluidic chip system. Time-lapse video recording was carried out (24 h, with a 20 min interval between frames) by using a confocal laser scanner microscope Zeiss LSM 900 (Carl Zeiss, Germany) equipped with an Ibidi incubation system (Ibidi, Germany) to monitor acquisition of green fluorescence by tumor cells as indicative of C16^+^ EV incorporation. For further evaluation of the uptake of the fluorescence within tumor cells, at the end of the experiment cells in the devices were fixed and stained with DAPI. Briefly, lateral chambers were washed with PBS 1X and then loaded with a fixation solution (Paraformaldehyde 2%, glutaraldehyde 1% in a 500 µL volume of PBS 1X, 50 µL/channel) with a 20 min incubation at room temperature in the dark. Chambers then were rinsed with PBS and stained with DAPI solution (20 µg/ml in 40 µL) by a 45 min incubation at room temperature in the dark. After wash with PBS, images were acquired on a confocal microscope Zeiss LSM 900 (Carl Zeiss GmbH, Jena, Germany) in Airyscan mode. Excitation light was obtained by diode lasers: 405 nm and 488 nm. Optical thickness according to objective used is 0.50 um with 63 × objective. Images have been treated and analyzed by the Zen Blue (3.2) software (Carl Zeiss GmbH, Jena Germany) and ImageJ (1.53) software (NIH, USA—http://imagej.nih.gov/ij).

### Exposure of tumor cells to eosinophils and eosinophil -derived EV

For co-culture of eosinophils with tumor cells, mouse B16.F10, TC1 or MCA205 and human A375P were cultured alone or with mouse and human Eo5 or Eo33 (respectively) at an Eo:Tumor cells ratio of 4:1. After 24 h incubation, eosinophils were removed by extensive washes with PBS and adherent tumor cells were collected for further analyses. To evaluate the eosinophil -derived EV-mediated activities on tumor cells, human or mouse Eo5-EV and Eo33-EV were directly administered to the culture medium of tumor cells. Approximately 20–40 × 10^6^ mouse Eo5-EV or Eo33-EV and 40–80 × 10^6^ hEo5-EV or hEo33-EV, corresponding to the amount of EV isolated from each mL of conditioned medium from 2 × 10^6^ eosinophils, were administered per well. Tumor cells were cultured 24 h at 37 °C in 5% CO_2_ and subsequently harvested and employed for analyses.

### Cell proliferation assays

The anti-proliferative capacity of eosinophil-derived EV was assessed by CellTiter 96 AQueous One Solution Cell Proliferation Assay (MTS assay; Promega) and by crystal violet staining. For MTS assay, one thousand tumor cells in 200 µL were let to adhere in flat 96-well plates. Eo5-EV or Eo33-EV (20–40 × 10^6^ per well) were added to the culture. Controls consisted in tumor cells cultured alone, without added EV. Each experimental condition was performed in triplicate. After various times, 20 µL of MTS reagent [3-(4,5-dimethylthiazol-2-yl)-5-(3-carboxymethoxyphenyl)-2-(4-sulfophenyl)-2H-tetrazolium] were added to each well. Plates were incubated for 1 h at 37°C in the dark. Absorbance at 490 nm was then determined in a spectrophotometer. For crystal violet assay, 1 × 10^5^ A375P and 7 × 10^4^ B16.F10, TC1, MCA205 were seeded in 6-well plates (Corning Inc.). Human or mouse Eo5-EV, Eo33-EV or half amount of Eo33-EV (diluted 1:2 to titrate Eo33-EV to the same amount as Eo5-EV) were directly administered to the culture medium of tumor cells. Tumor cells cultured alone, without added EV were used as control conditions. After incubation at 37 °C in 5% CO_2_ for 48 h (A375P, B16.F10) or 72 h (TC1, MCA205), adherent tumor cells were stained in 0,1% crystal violet (dissolved in 20% ethanol). For direct anti-proliferative capacity of human eosinophils against melanoma cells, A375P (1 × 10^5^) were co-cultured with 3 × 10^5^ hEo5 or hEo33 in 6-well plates (Corning Inc.) for 24 h (Eo:Tumor cells ratio, 3:1). At the end of the incubation, eosinophils were washed away and adherent tumor cells were stained in 0,1% crystal violet. Crystal violet-stained tumor cell images were captured in phase contrast light (20–40, 4X objective) and quantitative analysis of tumor cell covered area was performed with ImageJ Software (Particle analysis plugin). Each image was subjected to manual thresholding and particle analysis was performed on each thresholded image. This process allow to detect each tumor cell as a Region of Interest object (ROI). The sum of all ROIs of each image constitutes the total covered area (µm^2^) of proliferating tumor cells. The percentage of covered area is referred to the ratio between the detected area of cells in an image and the maximum theoretical area the cells can occupy in that image. The latter is expressed in µm^2^ and is calculated by multiplicating the X axis size (in µm) to Y axis size of the image (in µm).

### Annexin V apoptosis assay

Eosinophil-mediated cytotoxicity against tumor cells was evaluated as previously described [21].

Briefly, B16.F10 melanoma cells were labeled with the PKH26 Red fluorescent Cell Linker (Sigma) and then seeded in 96 wells U-bottomed plates (5 × 10^4^/well in 100 µL) in the presence of Eo5 or Eo33 (2.5 × 10^6^ in 100 µL; 50:1 E:T ratio). Were indicated, Eo5 and Eo33 were pre-incubated for 4 h with, respectively, 2 µM and 8 µM GW4869, which was further added during the co-culture with tumor cells. After 5 h incubation, tumor cells were stained with Annexin-V (e-Bioscience, Thermo Fisher Scientific) and analyzed by flow cytometry. Apoptosis of target tumor cells was calculated as the percentage of Annexin-V^+^ cells among gated PKH26^+^ population.

### Tumor spheroid formation

Multicellular tumor spheroids from murine B16.F10 melanoma, MCA205 fibrosarcoma, TC1 adenocarcinoma and human A375P melanoma cells were generated in flat ultralow-attachment surface 96-well plates (Corning Inc.). Five hundred tumor cells were cultured in 200 µL of medium alone or with added eosinophil-derived EV (20–40 × 10^6^ mouse Eo5-EV or Eo33-EV per well or 40–80 × 10^6^ hEo5-EV or hEo33-EV per well). Visible channel microphotographs were generated at different time points by using an EVOS-FL microscope (Life Technologies). Up to four regions were acquired per each spheroid well. The acquired replicate images were then subjected to particle analysis quantification by using the homonym ImageJ plugin. An optimal thresholding algorithm was chosen for each region, depending on visible light dispersion and distribution. Spheroid area was calculated and used as representative morphometric parameter.

### Cell cycle assay

Mouse and human tumor cells were plated (5 × 10^4^/well) in 6-well plates and incubated for 24 h with the corresponding mouse and human Eo5-EV or Eo33-EV (30–60 × 10^6^ mouse Eo5-EV or Eo33-EV per well or 40–80 × 10^6^ hEo5-EV or hEo33-EV per well). At the end of incubation, tumor cells were harvested, washed with PBS and resuspended in PBS supplemented with 0,1% Glucose. Tumor cells were permeabilized in 1 mL of ice-cold 70% ethanol and kept at + 4 °C. After 24 h, samples were washed and resuspended in 0.5 mL FxCycle™ PI/RNase Staining Solution (Thermo Fisher Scientific). Cells were incubated for 30 min at room temperature in the dark and then analyzed by flow cytometry for DNA content.

### Wound healing scratch assay

B16.F10 melanoma cells were seeded in 6-well plates and grown until 90–95% confluence, after which they were serum-starved in medium containing no FBS for 18 h in the absence (CTR) or presence of Eo5-EV or Eo33-EV (70–140 × 10^6^ per well). The following day, cells were scratched using a 200 μL sterile tip, washed with 1 mL of serum free DMEM to eliminate detached cells and cultured in fresh complete DMEM at 37 °C in 5% CO_2_. Images were acquired at various time points by using EVOS-FL fluorescence microscope and analyzed using ImageJ software to quantify the cell free area. Where indicated, EV were administered also after scratching the tumor cell monolayer.

### Morphometric analysis of tumor cells

To test the capacity of eosinophil-derived EV to affect tumor cell morphology, TC1 and B16.F10 cells (3 × 10^5^ cells in 2 mL) were let to adhere in 6 well glass bottom plates (Cellvis) then exposed to purified Eo5-EV, Eo33-EV (70–140 × 10^6^ mouse Eo5-EV or Eo33-EV per well) or cultured alone. Time lapse was performed by using the confocal laser scanner microscope Zeiss LSM 900 (Carl Zeiss, Germany) equipped with an Ibidi incubation system (Ibidi, Germany) for CO_2_ and temperature control. Frames were captured every 20 min (for a total of 140 min.) by employing an Axiocam 702 camera (Carl Zeiss, Germany), using a 10X objective. Excitation light was obtained by diode lasers: 405, 488, 561 and 640 nm. Optical thickness vary according to objective used from 0.50 mm with 20 × objective to 0.20 mm with 63 × objective. Acquired images were analyzed by the Zen Blue (3.2) software (Carl Zeiss, Germany) and morphological parameters (perimeter and Feret’s diameter) of tumor cells were evaluated by ImageJ software at representative time points extracted from the time lapse recording.

### E-Cadherin expression by flow cytometry

For the evaluation of E-Cadherin expression on cancer cell surface, mouse tumor cells (B16.F10 and TC1) were labeled with PKH26 Red fluorescent Cell Linker (Sigma) prior to co-culture with Eo5 or Eo33 and then stained with fluorescent anti-mouse/human CD324 (clone DECMA-1; Biolegend). E-Cadherin expression on tumor cells was evaluated by gating on PKH26^+^ cells.

### In vivo* pulmonary metastasis assay*

Five- to 7-week-old female C57BL/6 mice were purchased from ENVIGO Laboratories (Italy) and housed in the Istituto Superiore di Sanità animal facilities. For evaluation of lung metastasis formation, adherent sub-confluent B16.F10 melanoma cells were exposed to Eo5-EV, Eo33-EV (200–400 × 10^6^ EV), or cultured alone. After 24 h, untreated, Eo5-EV treated and Eo33-EV treated melanoma cells were harvested and injected in the tail vein (2 × 10^5^ in 200 µl PBS). At day 14, mice were sacrificed and lungs were inspected for metastatic foci count.

### Transmission *electron* microscopy

For transmission electron microscopy, Eo5 and Eo33 were fixed in 2.5% glutaraldehyde in 0.1 M cacodylate buffer, pH 7.4. Following washes in cacodylate buffer, cells were post-fixed in 1% OsO_4_ in the same buffer and further washed with 0.1 M cacodylate. Cells were then dehydrated in ethanol gradient from 50 to 100% (v/v) and embedded in Agar 100 resin (Agar Scientific) at 65°C for 48 h. Ultrathin sections were obtained by an ultra-microtome and collected on 200-mesh grids, counterstained with uranyl acetate for 10 min and lead citrate for further 10 min. Samples were observed in a Philips 208 s transmission electron microscope at 100 kW (Philips). For negative staining electron microscopy, a drop of each sample of EV, prepared as described above, was placed onto carbon-coated 400 mesh grids for 1 min and allow to adsorb. After blotting off excess of sample, 10 μL drop of 2% aqueous solution of sodium phosphotungstate were placed onto the grid for 20 s. Excess of staining were blotted off with filter paper, grids were allowed to air dry and observed at a Philips 208 electron microscope.

### Western blot

Western blot analysis was performed according to standard procedures. Mouse and human Eo5 and Eo33 -derived EV, and the respective producing cells, were resuspended in sample buffer with freshly added 50 μM DTT. Monoclonal antibodies (mAb) listed below were: mouse anti-Alix mAb (3A9 #MA183977, Thermo Fisher Scientific); mouse anti-TSG101 mAb (4A10 #GTX70255, GeneTex); human anti-CD81 mAb (1D6 #BTMC a-184 7 T, Clinisciences); mouse and human anti-Calnexin policlonal antibody (pAb) (#NB100-1974, NovusBio); mouse anti-GM130 mAb (35/GM130 #610,822, BD). Protein concentrations were measured with BCA Protein Assay (Thermo Fisher Scientific). Eight μg of each sample were loaded in 10% gel (SureCast Acrylamide Solution (40%), Invitrogen), and transferred to nitrocellulose membrane (Amersham). Images were acquired by using FluorChem (Protein Simple) and bands were quantified by densitometry using ImageJ software.

### Real time qPCR

Total RNA was extracted from tumor cells by using TRIsure reagent (Bioline). mRNA was reverse transcribed by means of Tetro cDNA Synthesis Kit (Bioline). Quantitative real time PCR (qPCR) with forward and reverse primers (Eurofins Genomics) was performed using Sensimix Plus SYBR Kit containing the fluorescent dye SYBR Green (Bioline) and by means of an ABI 7500 Real-time PCR system (Applied Biosystems, Thermo Fisher Scientific). The conditions of real time qPCR reaction were given as follows: 15 s at 95 °C, 30 s at 60 °C and 45 s at 72° for 40 cycles. Triplicates were performed for each experimental point. Quality and specificity of amplicons in each sample were detected by dissociation curve analysis. For mRNA expression quantitation, threshold cycle (Ct) values were determined by the sequence detection system software (Applied Biosystems). Data were normalized to HPRT housekeeping gene (2-∆Ct method). Reverse and forward primers used are listed in Table S1.

### RNA sequencing of eosinophils and derived EV

RNA Sequencing (RNA-Seq) was performed in collaboration with the Immunology Unit of University of Palermo. RNA was extracted from Eo5 and Eo33 and from Eo5-EV and Eo33-EV. Two experimental replicates for each sample (Eo5, Eo33, Eo5-EV and Eo33-EV) were prepared. Quality and quantity of extracted RNA from each sample were measured by the use of the spectrophotometer NanoDrop 1000 (Myco Instumentation). For library preparation, a low PCR barcode kit (cod. SQK-PBK004, Nanopore Technologies) was employed. To amplify all types of RNA molecules present in the samples, 280 to 1000 ng of total RNA extracted from eosinophils or their corresponding EV, respectively, were reverse transcribed into cDNA using the LunaScript RT SuperMix Master Mix (New England Biolabs, USA), following the manufacturer's protocol. This master mix contains random hexamer and poly-dT primers, ensuring even coverage across the length of the RNA targets. Reverse transcribed samples were then purified using AmpureXP beads (Beckman Coulter, USA), following the manufacturer’s instructions, and eluted in 49 μL of nuclease-free water. To determine the amount of cDNA present in each sample, Bioanalyzer 2100 (Agilent, USA) was used with the Agilent High Sensitivity DNA Kit (Agilent, USA). The library for sequencing was prepared according to the low PCR barcode (SQK-PBK004) protocol version PBK_9073_v1_revR_14August2019, starting from the End-prep step. Since the samples did not reach the suggested 100 femto-moles by the Nanopore protocol for library preparation, the entire volume (48 μL) was mixed with 3.5 μL of Ultra II End-prep reaction buffer and 3 μL of Ultra II End-prep enzyme mix (New England Biolabs, USA), resulting in a final volume of 54.5 μL. Next, to increase the concentration and to add a unique barcode sequence to each sample, an adapter ligation step was followed by an amplification step using barcoded primers and the enzymes as well as Thermal Profile recommended in the protocol. After amplification, each library was purified using AmpureXP beads. To determine the library molarity, the samples were analyzed on the Bioanalyzer 2100 using the DNA 12000 kit, following the manufacturer’s instructions. The libraries were then pooled into a total of 50 femto-moles in 10 μL of 10 mM Tris–HCl pH 8.0 with 50 mM NaCl. This pooled library was loaded onto the MinION flow cell version R9.4.1 FLO-MIN106 and sequenced through MinION Mk1B nanopores platform (Nanopore, product code MIN-101B), after the adapter ligation step. The MinION sequencer was connected to a PC, and MinKNOW software version 22.12.7 was used to sequence the libraries and perform base-calling of the generated reads, resulting in fast5 and fastq files, respectively.

### Bionformatic analysis of bulk RNA-Seq data

The produced fastq files were sent to IFOM/Cogentech SRL company service for Bioinformatic analysis. Fastq files were processed by nfcore/nanoseq pipeline (https://nf-co.re/nanoseq) by using the Ensemble version 109 for both fasta (ftp://ftp.ensembl.org/pub/release-109/fasta/mus_musculus/dna/Mus_musculus.GRCm39.dna.primary_assembly.fa) and gft (ftp://ftp.ensembl.org/pub/release109/gtf/mus_musculus/Mus_musculus.GRCm39.109.gtf) files. The following parameters were employed: input sample_sheet, protocol cDNA, skip_demultiplexing, skip_fusion_analysis, skip_differential_analysis. Pipeline was used for quality control, alignment, quality control of alignment, reconstruction and quantification of genes and transcripts. FeatureCount and StringTie tools were used for transcript quantification. DexSeq (|log2FC|> 0.58) was employed for the differential analysis. For normalization, Upper Quartile Normalization was employed. These experimental data are publicly available in the NCBI SRA Public Database (Bioproject accession no. PRJNA1041844). RNA sequencing process identified 149,223 transcripts in our samples (provided as an Excel file dataset), including protein coding RNAs (mRNAs), miRNAs, long noncoding RNAs (lncRNAs), ribosomal RNAs (rRNAs), pseudogenes, mitochondrial RNAs (mtRNAs) and other unprocessed transcripts. To exclusively select mRNAs from our dataset, we selected the protein coding transcripts in the “Transcript-biotype” field, resulting in 58,811 selected mRNAs. The base-2 logarithmic expression values of the two replicates in each experimental condition (Eo5, Eo33, Eo5-EV and Eo33-EV) were converted into exponential values. These values were used to extrapolate mRNAs differentially expressed in Eo33-EV and Eo5-EV. To this end, we cross-filtered transcripts by selecting values > 0 in each Eo5-EV and Eo33-EV replicate as well as in their producing cell (Eo5 or Eo33, respectively). We computed the mean fold change (FC) values between Eo33-EV with respect to Eo5-EV and significance by *t* test. Up-regulated mRNAs in Eo33-EV were computed by selecting > 2 FC-values and < 0.05 *P*-values, while up-regulated mRNAs in Eo5-EV were calculated by selecting < 0.5 FC-values and < 0.05 *P*-values. This cross-filtering process yielded 132 transcripts whose expression is significantly higher in Eo33-EV with respect to Eo5-EV and 88 transcripts whose expression is significantly higher in Eo5-EV compared to Eo33-EV. The differential expression of the clustered genes was visually plotted as heatmaps by using Orange Data Mining software (https://orangedatamining.com). Tumor suppressor genes in the two Eo5-EV and Eo33-EV mRNA signatures were revealed by data mining employing the Candidate Cancer Gene Database (CCGD; http://ccgd-starrlab.oit.umn.edu/search.html) and Tumor Suppressor Gene Database (TSGene; https://bioinfo.uth.edu/TSGene/). To represent the differential expression of Eo5-EV and Eo33-EV genes in Volcano plot, we generated an Excel dataset grouping the two cluster of genes. The graph was obtained by exploiting the VolcaNoseR2 R tool, an R script web-based tool (https://huygens.science.uva.nl/VolcaNoseR2/). Specifically, negative base-10 logarithm from Eo-EV *P*-values and base-2 logarithm from Eo-EV mean FC values (*n* = 15,630 mRNAs) were used to obtain a Volcano plot displaying Eo5-EV and Eo33-EV transcripts. FC value threshold was set up between -1 and + 1, while the significance threshold was configured on 1.1 (namely, around the 0.05 p raw value). Finally, Gene Ontology (GO) analysis of biological processes enriched in the two Eo5-EV and Eo33-EV signatures was carried out through the GProfiler database webtool (https://biit.cs.ut.ee/gprofiler/gost). The significance threshold was calculated as False Discovery Rate (FDR) upon a < 0.05 *P*-value cut off.

### Statistical analysis

Statistical analyses were performed using GraphPad Prism Software (GraphPad, La Jolla, CA). One-way ANOVA analysis was performed to compare means among multiple groups, followed by an appropriate post-hoc test. Values were considered significant when the probability was below the 5% confidence level (*p* < 0.05).

### Supplementary Information

Supplementary Material 1.

Supplementary Material 2.

Supplementary Material 3. Movie S1. Time lapse of fluorescent Eo5-EV incorporation into B16.F10 cells. C16-FL-labeled Eo5 and B16 cells were cultured in a microfluidic chip as illustrated in Figure 2B and described in Materials and Methods. Time lapse video recording was carried out by acquiring 68 frames every 20 min (for a total of 1360 min) using the bright field and the green channel fluorescence. Numbers at the center of the movie show the elapsed time (h:min). Acquisition of frames was performed in the region indicated in Figure 2B.

Supplementary Material 4. Movie S2. Time lapse of fluorescent Eo33-EV incorporation into B16.F10 cells. C16-FL-labeled Eo33 and B16 cells were cultured in a microfluidic chip as illustrated in Figure 2B and described in Materials and Methods. Time lapse video recording was carried out by acquiring 68 frames every 20 min (for a total of 1360 min) using the bright field and the green channel fluorescence. Numbers at the center of the movie show the elapsed time (h:min). Acquisition of frames was performed in the region indicated in Figure 2B.

Supplementary Material 5. Movie S3. 3D reproduction of Z-stack acquisition of fluorescent Eo5-EV incorporation into B16.F10 cell in the microfluidic devices depicted in Figure 2B. The acquisition was performed in Airyscan mode by a 63X immersion-oil objective for a total of 26 slices with an optical thickness of 0.5 μm each. Excitation light was obtained by diode lasers: 405 nm and 488 nm.

Supplementary Material 6. Movie S4. 3D reproduction of Z-stack acquisition of fluorescent Eo33-EV incorporation into B16.F10 cells in the microfluidic devices depicted in Figure 2B. The acquisition was performed in Airyscan mode by a 63X immersion-oil objective for a total of 27 slices with an optical thickness of 0.5 μm each. Excitation light was obtained by diode lasers: 405 nm and 488 nm.

## Data Availability

The data supporting the findings of this study are available from the corresponding author upon reasonable request.

## Abbreviations

*CDKI*: Cyclin-dependent kinase inhibitors
*Eo33*: IL-33-activated eosinophils
*Eo5*: IL-5-stimulated eosinophils
*Eo33-EV*: Extracellular vesicles derived from IL-33-activated eosinophils
*Eo5-EV*: Extracellular vesicles derived from IL-5-stimulated eosinophils
*EMT*: Epithelial-to-mesenchymal transition
*EV*: Extracellular vesicles
*FGF-2*: Fibroblast Growth Factor 2
*GO*: Gene Ontology
*hEo33*: Eosinophils purified from the peripheral blood of healthy donors and cultured overnight with IL-33
*hEo5*: Eosinophils purified from the peripheral blood of healthy donors and cultured overnight with IL-5
*hEo33-EV*: Extracellular vesicles derived from hEo33
*hEo5-EV*: Extracellular vesicles derived from hEo5
*lncRNA*: Long non-coding RNA
*mRNA*: Messenger RNA
*MVB*: Multivesicular bodies
*NTA*: Nanoparticle tracking analysis
*PTPRJ*: Protein Tyrosine Phosphatase Receptor J
*qPCR*: Quantitative real time polymerase chain reaction
*RNA-**seq*: RNA sequencing
*TME*: Tumor microenvironment

## References

[1] Kalluri R, McAndrews KM. The role of extracellular vesicles in cancer. Cell. 2023;186:1610–26. 10.1016/j.cell.2023.03.010. 37059067 10.1016/j.cell.2023.03.010 PMC10484374

[2] Wei H, Chen Q, Lin L, Sha C, Li T, Liu Y, Yin X, Xu Y, Chen L, Gao W, Li Y, et al. Regulation of exosome production and cargo sorting. Int J Biol Sci. 2021;17:163–77. 10.7150/ijbs.53671. 33390841 10.7150/ijbs.53671 PMC7757038

[3] Gurung S, Perocheau D, Touramanidou L, Baruteau J. The exosome journey: from biogenesis to uptake and intracellular signalling. Cell Commun Signal. 2021;19:47. 10.1186/s12964-021-00730-1. 33892745 10.1186/s12964-021-00730-1 PMC8063428

[4] Ginini L, Billan S, Fridman E, Gil Z. Insight into Extracellular Vesicle-Cell Communication: From Cell Recognition to Intracellular Fate. Cells. 2022;11(9):1375. 10.3390/cells11091375. 35563681 10.3390/cells11091375 PMC9101098

[5] Wang S, Shi Y. Exosomes Derived from Immune Cells: The New Role of Tumor Immune Microenvironment and Tumor Therapy. Int J Nanomedicine. 2022;17:6527–50. 10.2147/IJN.S388604. 36575698 10.2147/IJN.S388604 PMC9790146

[6] Wang X, Huang R, Lu Z, Wang Z, Chen X, Huang D. Exosomes from M1-polarized macrophages promote apoptosis in lung adenocarcinoma via the miR-181a-5p/ETS1/STK16 axis. Cancer Sci. 2022;113:986–1001. 10.1111/cas.15268. 35092121 10.1111/cas.15268 PMC8898733

[7] Jiang H, Zhou L, Shen N, Ning X, Wu D, Jiang K, Huang X. M1 macrophage-derived exosomes and their key molecule lncRNA HOTTIP suppress head and neck squamous cell carcinoma progression by upregulating the TLR5/NF-κB pathway. Cell Death Dis. 2022;13:183. 10.1038/s41419-022-04640-z. 35210436 10.1038/s41419-022-04640-z PMC8873565

[8] Li X, Tang M. Exosomes released from M2 macrophages transfer miR-221-3p contributed to EOC progression through targeting CDKN1B. Cancer Med. 2020;9:5976–88. 10.1002/cam4.3252. 32590883 10.1002/cam4.3252 PMC7433826

[9] Yin Z, Ma T, Huang B, Lin L, Zhou Y, Yan J, Zou Y, Chen S. Macrophage-derived exosomal microRNA-501-3p promotes progression of pancreatic ductal adenocarcinoma through the TGFBR3-mediated TGF-β signaling pathway. J Exp Clin Cancer Res. 2019;38:310. 10.1186/s13046-019-1313-x. 31307515 10.1186/s13046-019-1313-x PMC6631643

[10] Mi X, Xu R, Hong S, Xu T, Zhang W, Liu M. M2 Macrophage-Derived Exosomal lncRNA AFAP1-AS1 and MicroRNA-26a Affect Cell Migration and Metastasis in Esophageal Cancer. Mol Ther Nucleic Acids. 2020;22:779–90. 10.1016/j.omtn.2020.09.035. 33230475 10.1016/j.omtn.2020.09.035 PMC7595846

[11] Song L, Luan B, Xu Q, Shi R, Wang X. microRNA-155-3p delivered by M2 macrophages-derived exosomes enhances the progression of medulloblastoma through regulation of WDR82. J Transl Med. 2022;20:13. 10.1186/s12967-021-03156-y. 34983581 10.1186/s12967-021-03156-y PMC8728908

[12] Wu F, Xie M, Hun M, She Z, Li C, Luo S, Chen X, Wan W, Wen C, Tian J. Natural Killer Cell-Derived Extracellular Vesicles: Novel Players in Cancer Immunotherapy. Front Immunol. 2021;12: 658698. 10.3389/fimmu.2021.658698. 34093547 10.3389/fimmu.2021.658698 PMC8176011

[13] Cai Z, Yang F, Yu L, Yu Z, Jiang L, Wang Q, Yang Y, Wang L, Cao X, Wang J. Activated T cell exosomes promote tumor invasion via Fas signaling pathway. J Immunol. 2012;188:5954–61. 10.4049/jimmunol.1103466. 22573809 10.4049/jimmunol.1103466

[14] Wang X, Xiang Z, Liu Y, Huang C, Pei Y, Zhi H, Wong WH, Wei H, Ng IO, Lee PP, Chan GC, et al. Exosomes derived from Vδ2-T cells control Epstein-Barr virus-associated tumors and induce T cell antitumor immunity. Sci Transl Med. 2020;12(563):eaaz3426. 10.1126/scitranslmed.aaz3426. 32998970 10.1126/scitranslmed.aaz3426

[15] Li L, Lu S, Liang X, Cao B, Wang S, Jiang J, Luo H, He S, Lang J, Zhu G. γδTDEs: An Efficient Delivery System for miR-138 with Anti-tumoral and Immunostimulatory Roles on Oral Squamous Cell Carcinoma. Mol Ther Nucleic Acids. 2019;14:101–13. 10.1016/j.omtn.2018.11.009. 30594069 10.1016/j.omtn.2018.11.009 PMC6307324

[16] Yang Z, Wang W, Zhao L, Wang X, Gimple RC, Xu L, Wang Y, Rich JN, Zhou S. Plasma cells shape the mesenchymal identity of ovarian cancers through transfer of exosome-derived microRNAs. Sci Adv. 2021;7(9):eabb0737. 10.1126/sciadv.abb0737. 33627414 10.1126/sciadv.abb0737 PMC7904265

[17] Munich S, Sobo-Vujanovic A, Buchser WJ, Beer-Stolz D, Vujanovic NL. Dendritic cell exosomes directly kill tumor cells and activate natural killer cells via TNF superfamily ligands. Oncoimmunology. 2012;1:1074–83. 10.4161/onci.20897. 23170255 10.4161/onci.20897 PMC3494621

[18] Xiao H, Lässer C, Shelke GV, Wang J, Rådinger M, Lunavat TR, Malmhäll C, Lin LH, Li J, Li L, Lötvall J. Mast cell exosomes promote lung adenocarcinoma cell proliferation - role of KIT-stem cell factor signaling. Cell Commun Signal. 2014;12:64. 10.1186/s12964-014-0064-8. 25311367 10.1186/s12964-014-0064-8 PMC4206705

[19] Varricchi G, Galdiero MR, Loffredo S, Lucarini V, Marone G, Mattei F, Marone G, Schiavoni G. Eosinophils: The unsung heroes in cancer? OncoImmunology. 2018;7(2):e1393134.10.1080/2162402X.2017.1393134. 29308325 10.1080/2162402X.2017.1393134 PMC5749653

[20] Mattei F, Andreone S, Marone G, Gambardella AR, Loffredo S, Varricchi G, Schiavoni G. Eosinophils in the Tumor Microenvironment. Adv Exp Med Biol. 2020;1273:1–28. 10.1007/978-3-030-49270-0_1. 33119873 10.1007/978-3-030-49270-0_1

[21] Andreone S, Spadaro F, Buccione C, Mancini J, Tinari A, Sestili P, Gambardella AR, Lucarini V, Ziccheddu G, Parolini I, Zanetti C, et al. IL-33 Promotes CD11b/CD18-Mediated Adhesion of Eosinophils to Cancer Cells and Synapse-Polarized Degranulation Leading to Tumor Cell Killing. Cancers (Basel). 2019;11(11):1664. 10.3390/cancers11111664. 31717819 10.3390/cancers11111664 PMC6895824

[22] Lucarini V, Ziccheddu G, Macchia I, La Sorsa V, Peschiaroli F, Buccione C, Sistigu A, Sanchez M, Andreone S, D’Urso MT, Spada M, et al. IL-33 restricts tumor growth and inhibits pulmonary metastasis in melanoma-bearing mice through eosinophils. OncoImmunology. 2017;6(6):e1317420. 10.1080/2162402X.2017.1317420 28680750 10.1080/2162402X.2017.1317420 PMC5486175

[23] Kienzl M, Hasenoehrl C, Valadez-Cosmes P, Maitz K, Sarsembayeva A, Sturm E, Heinemann A, Kargl J, Schicho R. IL-33 reduces tumor growth in models of colorectal cancer with the help of eosinophils. Oncoimmunology. 2020;9(1):1776059. 10.1080/2162402X.2020.1776059. 32923137 10.1080/2162402X.2020.1776059 PMC7458617

[24] Reichman H, Itan M, Rozenberg P, Yarmolovski T, Brazowski E, Varol C, Gluck N, Shapira S, Arber N, Qimron U, Karo-Atar D, et al. Activated Eosinophils Exert Antitumorigenic Activities in Colorectal Cancer. Cancer Immunol Res. 2019;7:388–400. 10.1158/2326-6066.CIR-18-0494. 30665890 10.1158/2326-6066.CIR-18-0494

[25] Gatault S, Legrand F, Delbeke M, Loiseau S, Capron M. Involvement of eosinophils in the anti-tumor response. Cancer Immunol Immunother. 2012;61:1527–34. 10.1007/s00262-012-1288-3. 22706380 10.1007/s00262-012-1288-3 PMC11029779

[26] Legrand F, Driss V, Delbeke M, Loiseau S, Hermann E, Dombrowicz D, Capron M. Human eosinophils exert TNF-α and granzyme A-mediated tumoricidal activity toward colon carcinoma cells. J Immunol. 2010;185:7443–51. 10.4049/jimmunol.1000446. 21068403 10.4049/jimmunol.1000446

[27] Munitz A, Bachelet I, Fraenkel S, Katz G, Mandelboim O, Simon HU, Moretta L, Colonna M, Levi-Schaffer F. 2B4 (CD244) is expressed and functional on human eosinophils. J Immunol. 2005;174:110–8. 15611233 10.4049/jimmunol.174.1.110

[28] Hollande C, Boussier J, Ziai J, Nozawa T, Bondet V, Phung W, Lu B, Duffy D, Paradis V, Mallet V, Eberl G, et al. Inhibition of the dipeptidyl peptidase DPP4 (CD26) reveals IL-33-dependent eosinophil-mediated control of tumor growth. Nat Immunol. 2019;20:257–64. 10.1038/s41590-019-0321-5. 30778250 10.1038/s41590-019-0321-5

[29] Mazzeo C, Cañas JA, Zafra MP, Rojas Marco A, Fernández-Nieto M, Sanz V, Mittelbrunn M, Izquierdo M, Baixaulli F, Sastre J, Del Pozo V. Exosome secretion by eosinophils: A possible role in asthma pathogenesis. J Allergy Clin Immunol. 2015;135:1603–13. 10.1016/j.jaci.2014.11.026. 25617225 10.1016/j.jaci.2014.11.026

[30] Cañas JA, Sastre B, Mazzeo C, Fernández-Nieto M, Rodrigo-Muñoz JM, González-Guerra A, Izquierdo M, Barranco P, Quirce S, Sastre J, Del Pozo V. Exosomes from eosinophils autoregulate and promote eosinophil functions. J Leukoc Biol. 2017;101:1191–9. 10.1189/jlb.3AB0516-233RR. 28096299 10.1189/jlb.3AB0516-233RR

[31] Cañas JA, Sastre B, Rodrigo-Muñoz JM, Fernández-Nieto M, Barranco P, Quirce S, Sastre J, Del Pozo V. Eosinophil-derived exosomes contribute to asthma remodelling by activating structural lung cells. Clin Exp Allergy. 2018;48:1173–85. 10.1111/cea.13122. 29451337 10.1111/cea.13122

[32] Cañas JA, Rodrigo-Muñoz JM, Del Pozo V. Isolation and Functional Aspects of Eosinophil-Derived Exosomes. Methods Mol Biol. 2021;2241:149–59. 10.1007/978-1-0716-1095-4_13. 33486735 10.1007/978-1-0716-1095-4_13

[33] Coscia C, Parolini I, Sanchez M, Biffoni M, Boussadia Z, Zanetti C, Fiani ML, Sargiacomo M. Generation, Quantification, and Tracing of Metabolically Labeled Fluorescent Exosomes. Methods Mol Biol. 2016;1448:217–35. 10.1007/978-1-4939-3753-0_16. 27317184 10.1007/978-1-4939-3753-0_16

[34] Barreca V, Boussadia Z, Polignano D, Galli L, Tirelli V, Sanchez M, Falchi M, Bertuccini L, Iosi F, Tatti M, Sargiacomo M, et al. Metabolic labelling of a subpopulation of small extracellular vesicles using a fluorescent palmitic acid analogue. J Extracell Vesicles. 2023;12: e12392. 10.1002/jev2.12392. 38072803 10.1002/jev2.12392 PMC10710952

[35] De Ninno A, Bertani FR, Gerardino A, Schiavoni G, Musella M, Galassi C, Mattei F, Sistigu A, Businaro L. Microfluidic Co-Culture Models for Dissecting the Immune Response in in vitro Tumor Microenvironments. J Vis Exp. 2021. 10.3791/61895. 33999026 10.3791/61895

[36] Kosaka N, Iguchi H, Yoshioka Y, Takeshita F, Matsuki Y, Ochiya T. Secretory mechanisms and intercellular transfer of microRNAs in living cells. J Biol Chem. 2010;285:17442–52. 10.1074/jbc.M110.107821. 20353945 10.1074/jbc.M110.107821 PMC2878508

[37] Boussadia Z, Lamberti J, Mattei F, Pizzi E, Puglisi R, Zanetti C, Pasquini L, Fratini F, Fantozzi L, Felicetti F, Fecchi K, et al. Acidic microenvironment plays a key role in human melanoma progression through a sustained exosome mediated transfer of clinically relevant metastatic molecules. J Exp Clin Cancer Res. 2018;37:245. 10.1186/s13046-018-0915-z. 30290833 10.1186/s13046-018-0915-z PMC6173926

[38] Andreone S, Gambardella AR, Mancini J, Loffredo S, Marcella S, La Sorsa V, Varricchi G, Schiavoni G, Mattei F. Anti-Tumorigenic Activities of IL-33: A Mechanistic Insight. Front Immunol. 2020;11: 571593. 10.3389/fimmu.2020.571593. 33329534 10.3389/fimmu.2020.571593 PMC7734277

[39] Carretero R, Sektioglu IM, Garbi N, Salgado OC, Beckhove P, Hämmerling GJ. Eosinophils orchestrate cancer rejection by normalizing tumor vessels and enhancing infiltration of CD8(+) T cells. Nat Immunol. 2015;16:609–17. 10.1038/ni.3159. 25915731 10.1038/ni.3159

[40] Efraim NB, A.H., and Levi-Schaffer, F. Roles of eosinophils in the modulation of angiogenesis. Chem Immunol Allergy. 2014;99:138–54. 10.1159/000353251. 24217607 10.1159/000353251

[41] Akuthota P, Carmo LA, Bonjour K, Murphy RO, Silva TP, Gamalier JP, Capron KL, Tigges J, Toxavidis V, Camacho V, Ghiran I, et al. Extracellular Microvesicle Production by Human Eosinophils Activated by “Inflammatory” Stimuli. Front Cell Dev Biol. 2016;4:117. 10.3389/fcell.2016.00117. 27833910 10.3389/fcell.2016.00117 PMC5081571

[42] Leal-Esteban LC, Fajas L. Cell cycle regulators in cancer cell metabolism. Biochim Biophys Acta Mol Basis Dis. 2020;1866: 165715. 10.1016/j.bbadis.2020.165715. 32035102 10.1016/j.bbadis.2020.165715

[43] Gatault S, Delbeke M, Driss V, Sarazin A, Dendooven A, Kahn JE, Lefèvre G, Capron M. IL-18 Is Involved in Eosinophil-Mediated Tumoricidal Activity against a Colon Carcinoma Cell Line by Upregulating LFA-1 and ICAM-1. J Immunol. 2015;195:2483–92. 10.4049/jimmunol.1402914. 26216891 10.4049/jimmunol.1402914

[44] Loh CY, Chai JY, Tang TF, Wong WF, Sethi G, Shanmugam MK, Chong PP, Looi CY. The E-Cadherin and N-Cadherin Switch in Epithelial-to-Mesenchymal Transition: Signaling, Therapeutic Implications, and Challenges. Cells. 2019;8(10):1118. 10.3390/cells8101118. 31547193 10.3390/cells8101118 PMC6830116

[45] Garcia MA, Nelson WJ, Chavez N. Cell-Cell Junctions Organize Structural and Signaling Networks. Cold Spring Harb Perspect Biol. 2018;10(4):a029181. 10.1101/cshperspect.a029181. 28600395 10.1101/cshperspect.a029181 PMC5773398

[46] Campbell K. Contribution of epithelial-mesenchymal transitions to organogenesis and cancer metastasis. Curr Opin Cell Biol. 2018;55:30–5. 10.1016/j.ceb.2018.06.008. 30006053 10.1016/j.ceb.2018.06.008 PMC6284102

[47] Mittal V. Epithelial Mesenchymal Transition in Tumor Metastasis. Annu Rev Pathol. 2018;13:395–412. 10.1146/annurev-pathol-020117-043854. 29414248 10.1146/annurev-pathol-020117-043854

[48] Ocaña OH, Córcoles R, Fabra A, Moreno-Bueno G, Acloque H, Vega S, Barrallo-Gimeno A, Cano A, Nieto MA. Metastatic colonization requires the repression of the epithelial-mesenchymal transition inducer Prrx1. Cancer Cell. 2012;22:709–24. 10.1016/j.ccr.2012.10.012. 23201163 10.1016/j.ccr.2012.10.012

[49] Tran HD, Luitel K, Kim M, Zhang K, Longmore GD, Tran DD. Transient SNAIL1 expression is necessary for metastatic competence in breast cancer. Cancer Res. 2014;74:6330–40. 10.1158/0008-5472.CAN-14-0923. 25164016 10.1158/0008-5472.CAN-14-0923 PMC4925010

[50] Stankic M, Pavlovic S, Chin Y, Brogi E, Padua D, Norton L, Massagué J, Benezra R. TGF-β-Id1 signaling opposes Twist1 and promotes metastatic colonization via a mesenchymal-to-epithelial transition. Cell Rep. 2013;5:1228–42. 10.1016/j.celrep.2013.11.014. 24332369 10.1016/j.celrep.2013.11.014 PMC3891470

[51] Veloso ES, de Carvalho BA, de Souza Silva FH, Ribeiro TS, Lima BM, Almeida CP, da Silva VHSR, Rocha SA, de Araújo Campos MR, Del Puerto HL, Ferreira E. Epithelial-mesenchymal transition inhibition by metformin reduces melanoma lung metastasis in a murine model. Sci Rep. 2022;12:17776. 10.1038/s41598-022-22235-8. 36273071 10.1038/s41598-022-22235-8 PMC9588059

[52] Lim JCW, Kwan YP, Tan MS, Teo MHY, Chiba S, Wahli W, Wang X. The Role of PPARβ/δ in Melanoma Metastasis. Int J Mol Sci. 2018;19(10):2860. 10.3390/ijms19102860. 30241392 10.3390/ijms19102860 PMC6213649

[53] Tian L, Li L, Xing W, Li R, Pei C, Dong X, Fu Y, Gu C, Guo X, Jia Y, Wang G, et al. IRGM1 enhances B16 melanoma cell metastasis through PI3K-Rac1 mediated epithelial mesenchymal transition. Sci Rep. 2015;5:12357. 10.1038/srep12357. 26202910 10.1038/srep12357 PMC4512008

[54] Revenco T, Nicodème A, Pastushenko I, Sznurkowska MK, Latil M, Sotiropoulou PA, Dubois C, Moers V, Lemaire S, de Maertelaer V, Blanpain C. Context Dependency of Epithelial-to-Mesenchymal Transition for Metastasis. Cell Rep. 2019;29:1458–1468.e1453. 10.1016/j.celrep.2019.09.081. 31693888 10.1016/j.celrep.2019.09.081

[55] Oh TI, Lee M, Lee YM, Kim GH, Lee D, You JS, Kim SH, Choi M, Jang H, Park YM, Shin HW, et al. PGC1α Loss Promotes Lung Cancer Metastasis through Epithelial-Mesenchymal Transition. Cancers (Basel). 2021;13(8):1772. 10.3390/cancers13081772. 33917757 10.3390/cancers13081772 PMC8068195

[56] Berr AL, Wiese K, Dos Santos G, Koch CM, Anekalla KR, Kidd M, Davis JM, Cheng Y, Hu YS, Ridge KM. Vimentin is required for tumor progression and metastasis in a mouse model of non-small cell lung cancer. Oncogene. 2023;42:2074–87. 10.1038/s41388-023-02703-9. 37161053 10.1038/s41388-023-02703-9 PMC10275760

[57] Lee MS, Lee J, Kim YM, Lee H. The metastasis suppressor CD82/KAI1 represses the TGF-β. Prostate. 2019;79:1400–11. 10.1002/pros.23837. 31212375 10.1002/pros.23837

[58] Cai YJ, Ma B, Wang ML, Chen J, Zhao FG, Zhou JD, Guo X, Zheng L, Xu CJ, Wang Y, He YB, et al. Impact of Nischarin on EMT regulators in breast cancer cell lines. Oncol Lett. 2020;20:291. 10.3892/ol.2020.12154. 33101485 10.3892/ol.2020.12154 PMC7576990

[59] Maziveyi M, Dong S, Baranwal S, Mehrnezhad A, Rathinam R, Huckaba TM, Mercante DE, Park K, Alahari SK. Exosomes from Nischarin-Expressing Cells Reduce Breast Cancer Cell Motility and Tumor Growth. Cancer Res. 2019;79:2152–66. 10.1158/0008-5472.CAN-18-0842. 30635277 10.1158/0008-5472.CAN-18-0842 PMC7204893

[60] Bereczki O, Ujfaludi Z, Pardi N, Nagy Z, Tora L, Boros IM, Balint E. TATA binding protein associated factor 3 (TAF3) interacts with p53 and inhibits its function. BMC Mol Biol. 2008;9:57. 10.1186/1471-2199-9-57. 18549481 10.1186/1471-2199-9-57 PMC2441632

[61] Seo J, Lozano MM, Dudley JP. Nuclear matrix binding regulates SATB1-mediated transcriptional repression. J Biol Chem. 2005;280:24600–9. 10.1074/jbc.M414076200. 15851481 10.1074/jbc.M414076200

[62] Hill AA, Riley PR. Differential regulation of Hand1 homodimer and Hand1-E12 heterodimer activity by the cofactor FHL2. Mol Cell Biol. 2004;24:9835–47. 10.1128/MCB.24.22.9835-9847.2004. 15509787 10.1128/MCB.24.22.9835-9847.2004 PMC525463

[63] Oshima M, Mimura J, Sekine H, Okawa H, Fujii-Kuriyama Y. SUMO modification regulates the transcriptional repressor function of aryl hydrocarbon receptor repressor. J Biol Chem. 2009;284:11017–26. 10.1074/jbc.M808694200. 19251700 10.1074/jbc.M808694200 PMC2670107

[64] Carpenter B, Hill KJ, Charalambous M, Wagner KJ, Lahiri D, James DI, Andersen JS, Schumacher V, Royer-Pokora B, Mann M, Ward A, et al. BASP1 is a transcriptional cosuppressor for the Wilms’ tumor suppressor protein WT1. Mol Cell Biol. 2004;24:537–49. 10.1128/MCB.24.2.537-549.2004. 14701728 10.1128/MCB.24.2.537-549.2004 PMC343806

[65] Wong DT, Kim JJ, Khalid O, Sun HH, Kim Y. Double edge: CDK2AP1 in cell-cycle regulation and epigenetic regulation. J Dent Res. 2012;91:235–41. 10.1177/0022034511420723. 21865592 10.1177/0022034511420723 PMC3275332

[66] Sampson C, Wang Q, Otkur W, Zhao H, Lu Y, Liu X, Piao HL. The roles of E3 ubiquitin ligases in cancer progression and targeted therapy. Clin Transl Med. 2023;13: e1204. 10.1002/ctm2.1204. 36881608 10.1002/ctm2.1204 PMC9991012

[67] Zhao BW, Sun SM, Xu K, Li YY, Lei WL, Li L, Liu SL, Ouyang YC, Sun QY, Wang ZB. FBXO34 Regulates the G2/M Transition and Anaphase Entry in Meiotic Oocytes. Front Cell Dev Biol. 2021;9: 647103. 10.3389/fcell.2021.647103. 33842473 10.3389/fcell.2021.647103 PMC8027338

[68] Li H, Zhang P, Liu C, Wang Y, Deng Y, Dong W, Yu Y. The Structure, Function and Regulation of Protein Tyrosine Phosphatase Receptor Type J and Its Role in Diseases. Cells. 2022;12(1):8. 10.3390/cells12010008. 36611803 10.3390/cells12010008 PMC9818648

[69] Hulea L, Nepveu A. CUX1 transcription factors: from biochemical activities and cell-based assays to mouse models and human diseases. Gene. 2012;497:18–26. 10.1016/j.gene.2012.01.039. 22306263 10.1016/j.gene.2012.01.039

[70] Cheng Z, Wei W, Wu Z, Wang J, Ding X, Sheng Y, Han Y, Wu Q. ARPC2 promotes breast cancer proliferation and metastasis. Oncol Rep. 2019;41:3189–200. 10.3892/or.2019.7113. 31002363 10.3892/or.2019.7113 PMC6488984

[71] Kim SY, Dunn IF, Firestein R, Gupta P, Wardwell L, Repich K, Schinzel AC, Wittner B, Silver SJ, Root DE, Boehm JS, et al. CK1epsilon is required for breast cancers dependent on beta-catenin activity. PLoS ONE. 2010;5: e8979. 10.1371/journal.pone.0008979. 20126544 10.1371/journal.pone.0008979 PMC2813871

[72] Grisaru-Tal S, Dulberg S, Beck L, Zhang C, Itan M, Hediyeh-Zadeh S, Caldwell J, Rozenberg P, Dolitzky A, Avlas S, Hazut I, et al. Metastasis-Entrained Eosinophils Enhance Lymphocyte-Mediated Antitumor Immunity. Cancer Res. 2021;81:5555–71. 10.1158/0008-5472.CAN-21-0839. 34429328 10.1158/0008-5472.CAN-21-0839

[73] Jung I, Shin S, Baek MC, Yea K. Modification of immune cell-derived exosomes for enhanced cancer immunotherapy: current advances and therapeutic applications. Exp Mol Med. 2024;56:19–31. 10.1038/s12276-023-01132-8. 38172594 10.1038/s12276-023-01132-8 PMC10834411

