# Supplementary material for: IL-33 stimulates the anticancer activities of eosinophils through extracellular vesicle-driven reprogramming of tumor cells
Source: J Exp Clin Cancer Res. 2024 Jul 27;43:209. doi: 10.1186/s13046-024-03129-1 (PMC11282757; doi:10.1186/s13046-024-03129-1)
Supplement: Supplementary file 1 — Supplementary Material 1. [file 13046_2024_3129_MOESM1_ESM.pptx]

## Slide 1
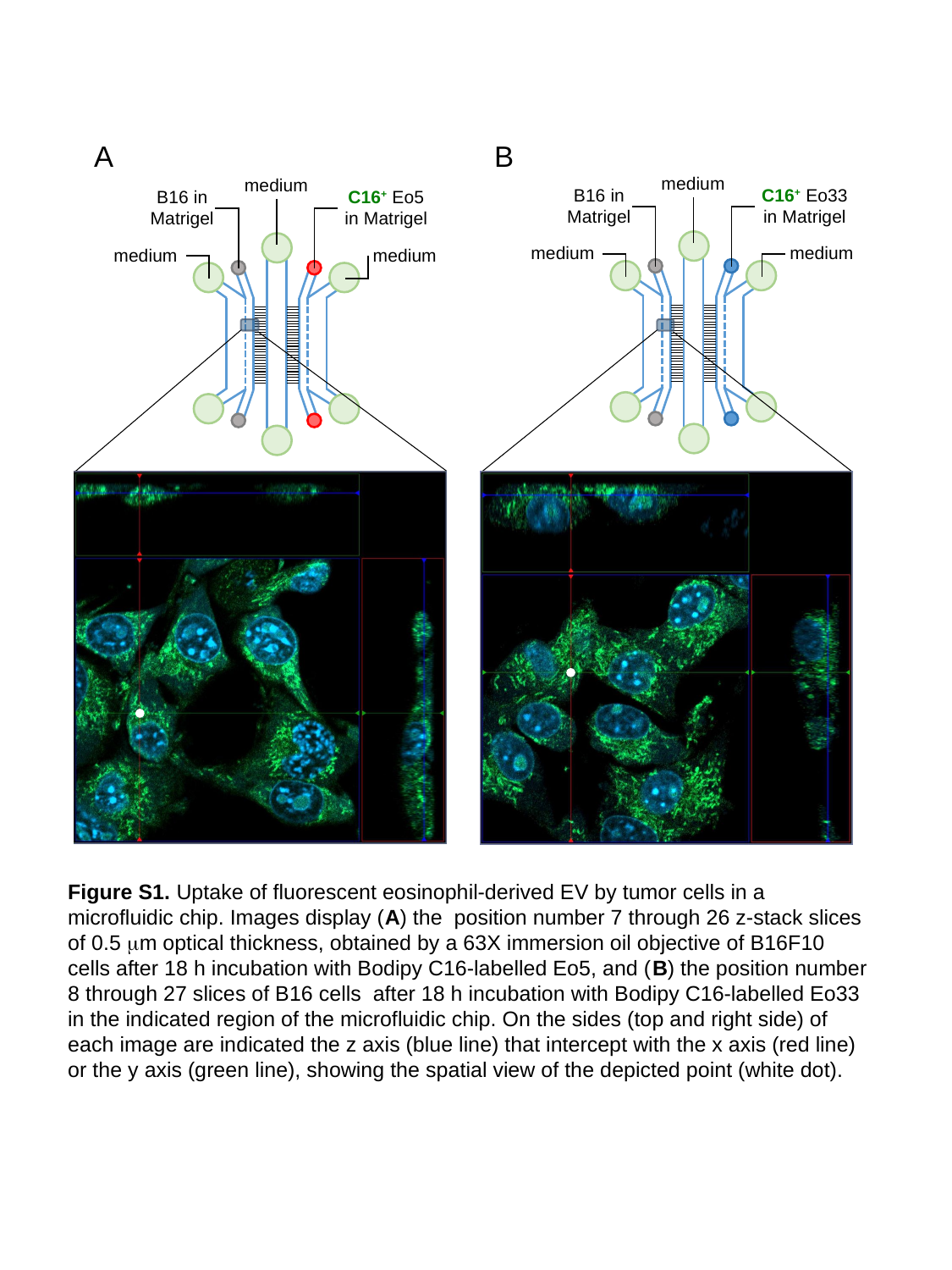

A
B
medium
B16 in Matrigel
C16+ Eo33
in Matrigel
medium
medium
medium
B16 in Matrigel
C16+ Eo5
in Matrigel
medium
medium
Figure S1. Uptake of fluorescent eosinophil-derived EV by tumor cells in a microfluidic chip. Images display (A) the position number 7 through 26 z-stack slices of 0.5 μm optical thickness, obtained by a 63X immersion oil objective of B16F10 cells after 18 h incubation with Bodipy C16-labelled Eo5, and (B) the position number 8 through 27 slices of B16 cells  after 18 h incubation with Bodipy C16-labelled Eo33 in the indicated region of the microfluidic chip. On the sides (top and right side) of each image are indicated the z axis (blue line) that intercept with the x axis (red line) or the y axis (green line), showing the spatial view of the depicted point (white dot).

## Slide 2
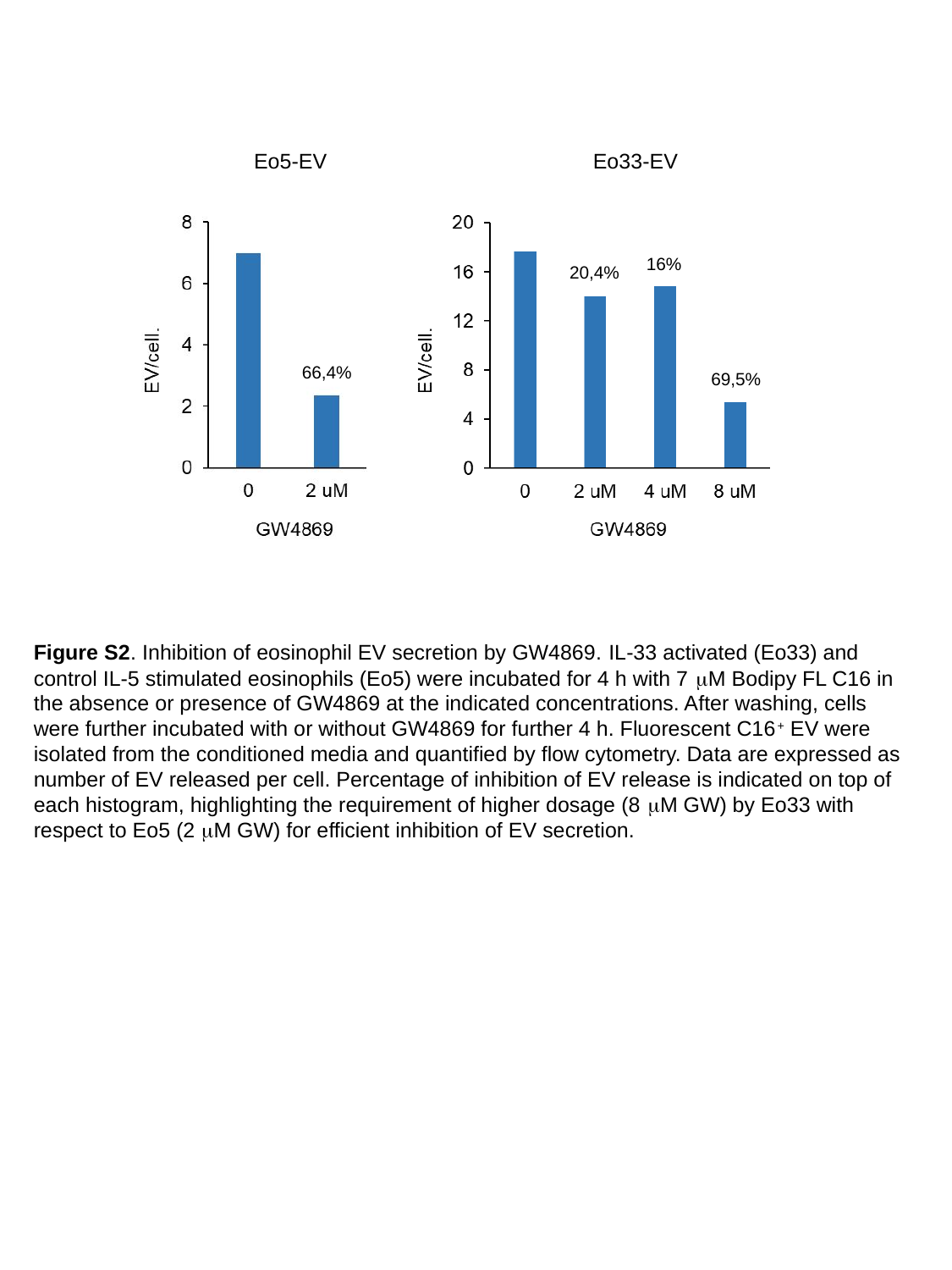

Eo5-EV
Eo33-EV
16%
20,4%
66,4%
69,5%
Figure S2. Inhibition of eosinophil EV secretion by GW4869. IL-33 activated (Eo33) and control IL-5 stimulated eosinophils (Eo5) were incubated for 4 h with 7 μM Bodipy FL C16 in the absence or presence of GW4869 at the indicated concentrations. After washing, cells were further incubated with or without GW4869 for further 4 h. Fluorescent C16+ EV were isolated from the conditioned media and quantified by flow cytometry. Data are expressed as number of EV released per cell. Percentage of inhibition of EV release is indicated on top of each histogram, highlighting the requirement of higher dosage (8 μM GW) by Eo33 with respect to Eo5 (2 μM GW) for efficient inhibition of EV secretion.

## Slide 3
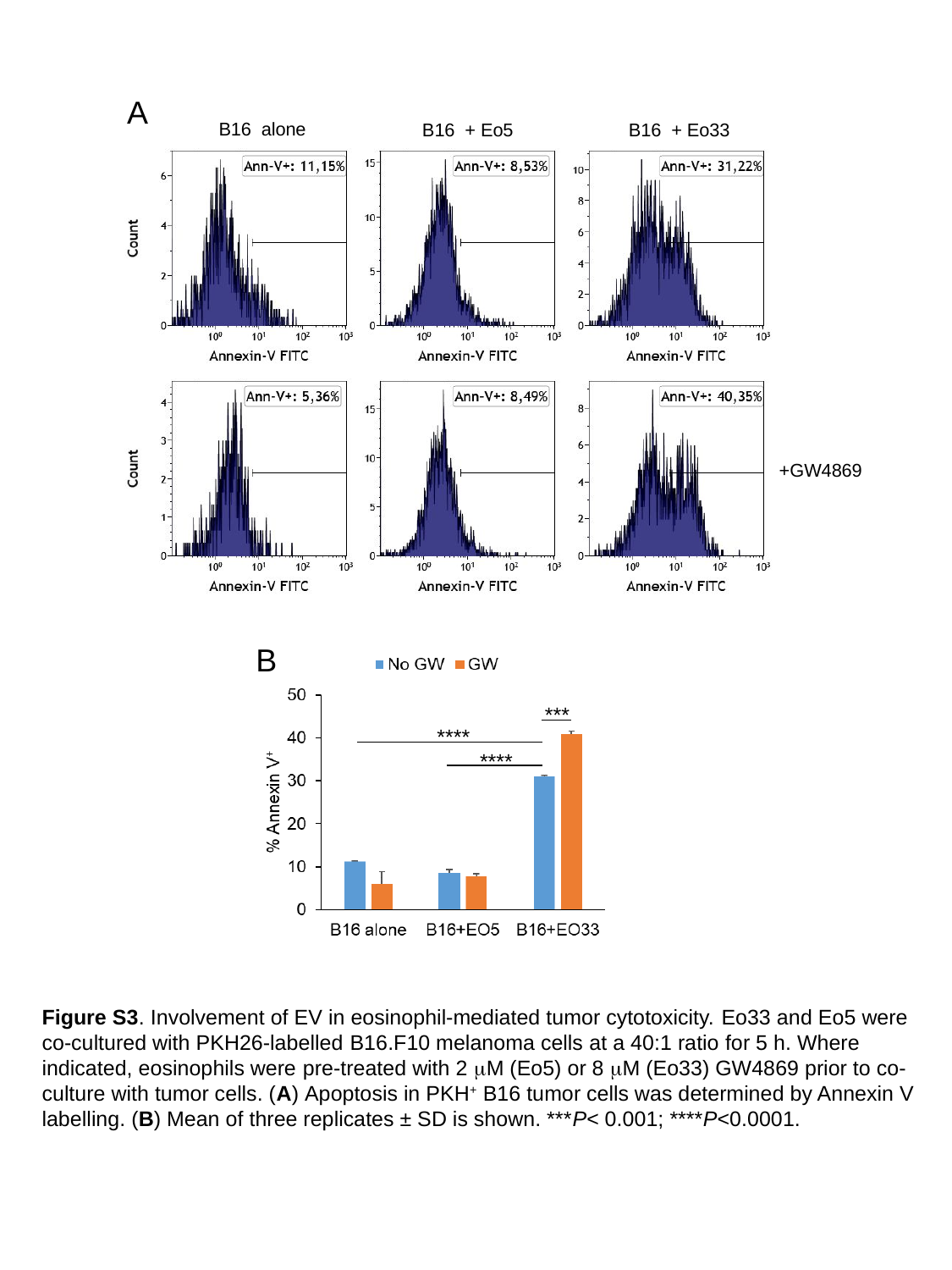

A
B16 alone
B16 + Eo5
B16 + Eo33
+GW4869
B
***
****
****
Figure S3. Involvement of EV in eosinophil-mediated tumor cytotoxicity. Eo33 and Eo5 were co-cultured with PKH26-labelled B16.F10 melanoma cells at a 40:1 ratio for 5 h. Where indicated, eosinophils were pre-treated with 2 μM (Eo5) or 8 μM (Eo33) GW4869 prior to co-culture with tumor cells. (A) Apoptosis in PKH+ B16 tumor cells was determined by Annexin V labelling. (B) Mean of three replicates ± SD is shown. ***P< 0.001; ****P<0.0001.

## Slide 4
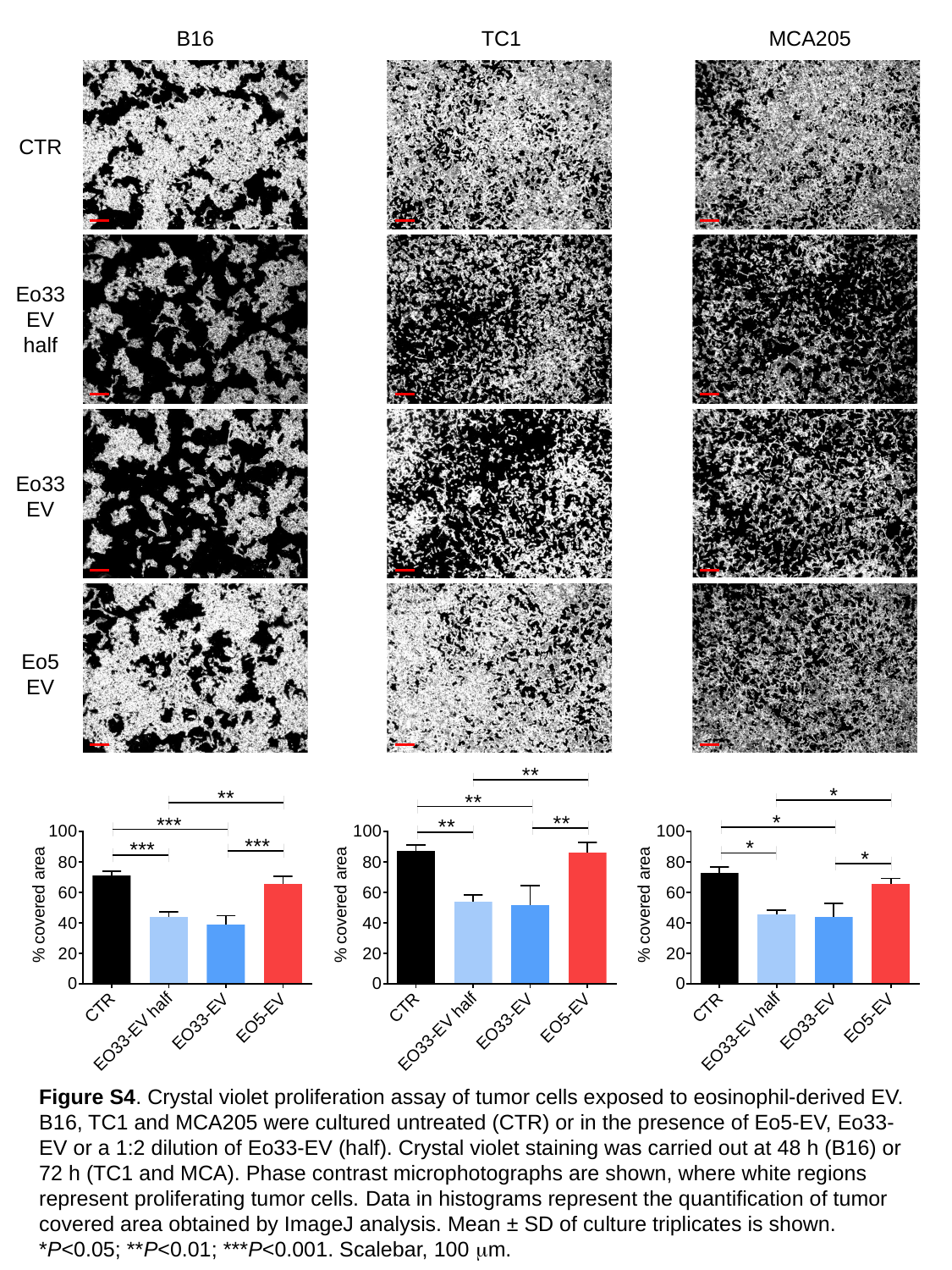

B16
TC1
MCA205
CTR
Eo33
EV
half
Eo33
EV
Eo5
EV
Figure S4. Crystal violet proliferation assay of tumor cells exposed to eosinophil-derived EV. B16, TC1 and MCA205 were cultured untreated (CTR) or in the presence of Eo5-EV, Eo33-EV or a 1:2 dilution of Eo33-EV (half). Crystal violet staining was carried out at 48 h (B16) or 72 h (TC1 and MCA). Phase contrast microphotographs are shown, where white regions represent proliferating tumor cells. Data in histograms represent the quantification of tumor covered area obtained by ImageJ analysis. Mean ± SD of culture triplicates is shown. *P<0.05; **P<0.01; ***P<0.001. Scalebar, 100 mm.

## Slide 5
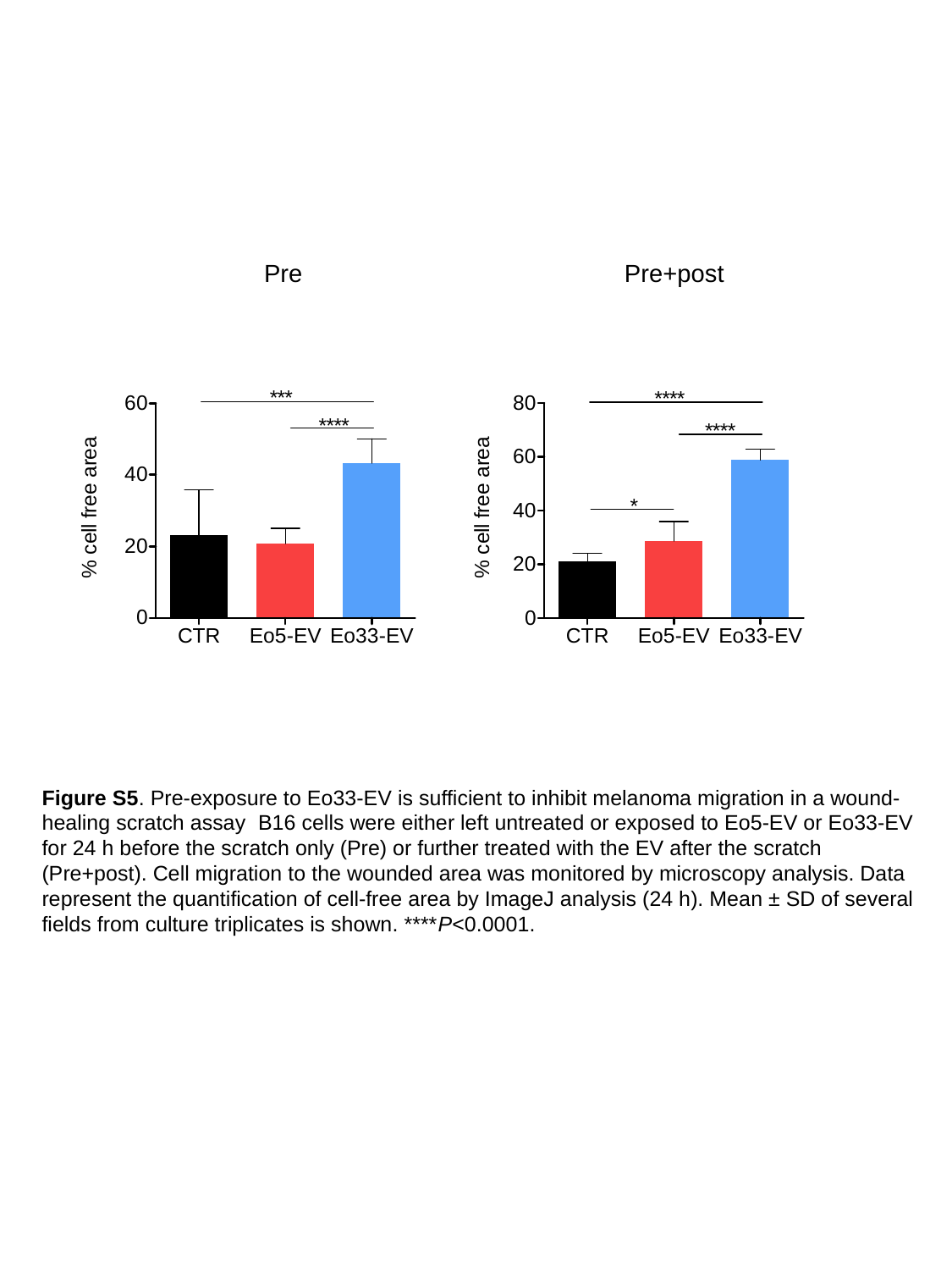

Pre
Pre+post
Figure S5. Pre-exposure to Eo33-EV is sufficient to inhibit melanoma migration in a wound-healing scratch assay B16 cells were either left untreated or exposed to Eo5-EV or Eo33-EV for 24 h before the scratch only (Pre) or further treated with the EV after the scratch (Pre+post). Cell migration to the wounded area was monitored by microscopy analysis. Data represent the quantification of cell-free area by ImageJ analysis (24 h). Mean ± SD of several fields from culture triplicates is shown. ****P<0.0001.

## Slide 6
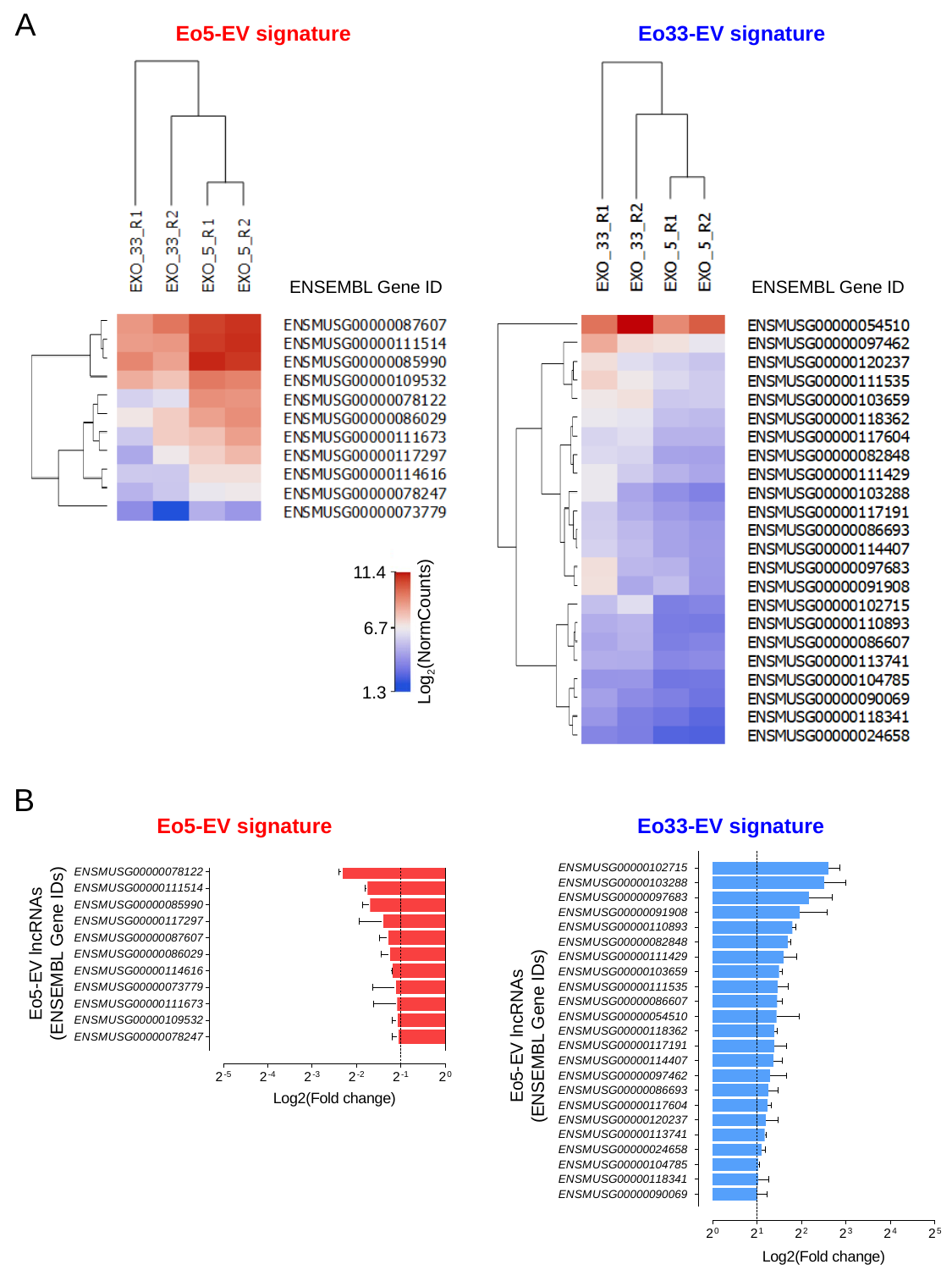

A
Eo5-EV signature
Eo33-EV signature
ENSEMBL Gene ID
ENSEMBL Gene ID
11.4
6.7
1.3
Log2(NormCounts)
B
Eo5-EV signature
Eo33-EV signature
Eo5-EV lncRNAs
(ENSEMBL Gene IDs)
Eo5-EV lncRNAs
(ENSEMBL Gene IDs)

## Slide 7
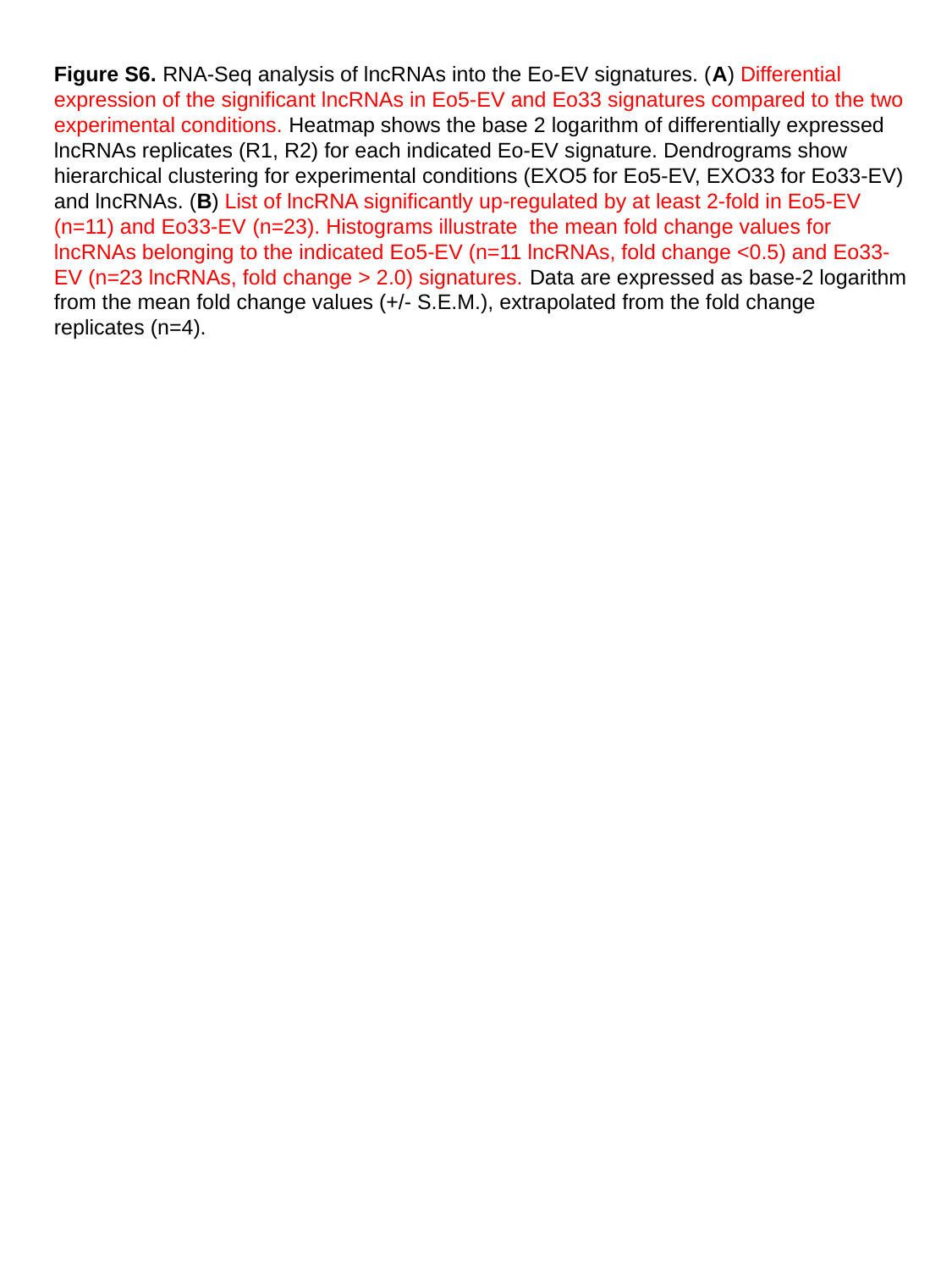

Figure S6. RNA-Seq analysis of lncRNAs into the Eo-EV signatures. (A) Differential expression of the significant lncRNAs in Eo5-EV and Eo33 signatures compared to the two experimental conditions. Heatmap shows the base 2 logarithm of differentially expressed lncRNAs replicates (R1, R2) for each indicated Eo-EV signature. Dendrograms show hierarchical clustering for experimental conditions (EXO5 for Eo5-EV, EXO33 for Eo33-EV) and lncRNAs. (B) List of lncRNA significantly up-regulated by at least 2-fold in Eo5-EV (n=11) and Eo33-EV (n=23). Histograms illustrate the mean fold change values for lncRNAs belonging to the indicated Eo5-EV (n=11 lncRNAs, fold change <0.5) and Eo33-EV (n=23 lncRNAs, fold change > 2.0) signatures. Data are expressed as base-2 logarithm from the mean fold change values (+/- S.E.M.), extrapolated from the fold change replicates (n=4).

## Slide 8
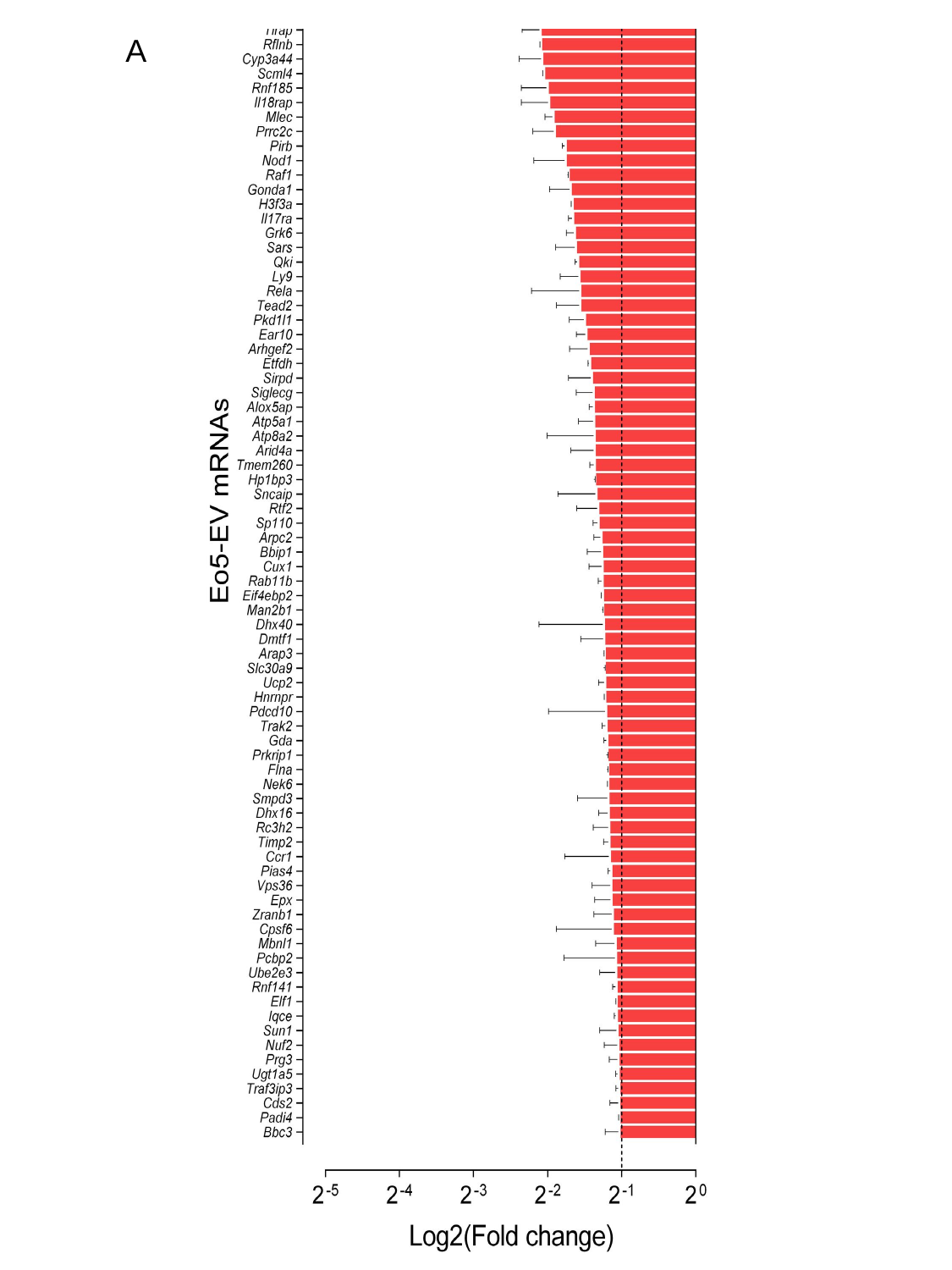

A

## Slide 9
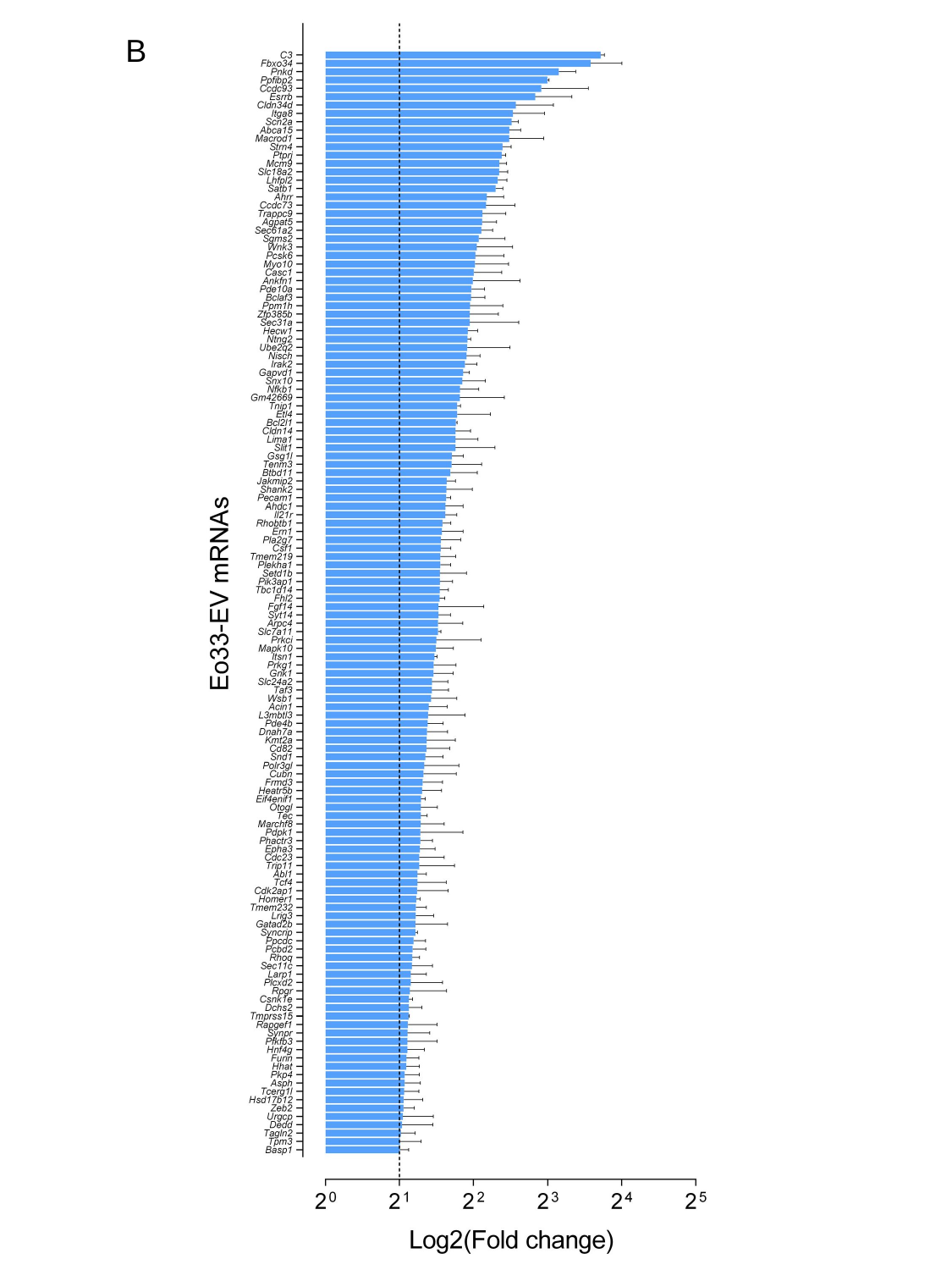

B

## Slide 10
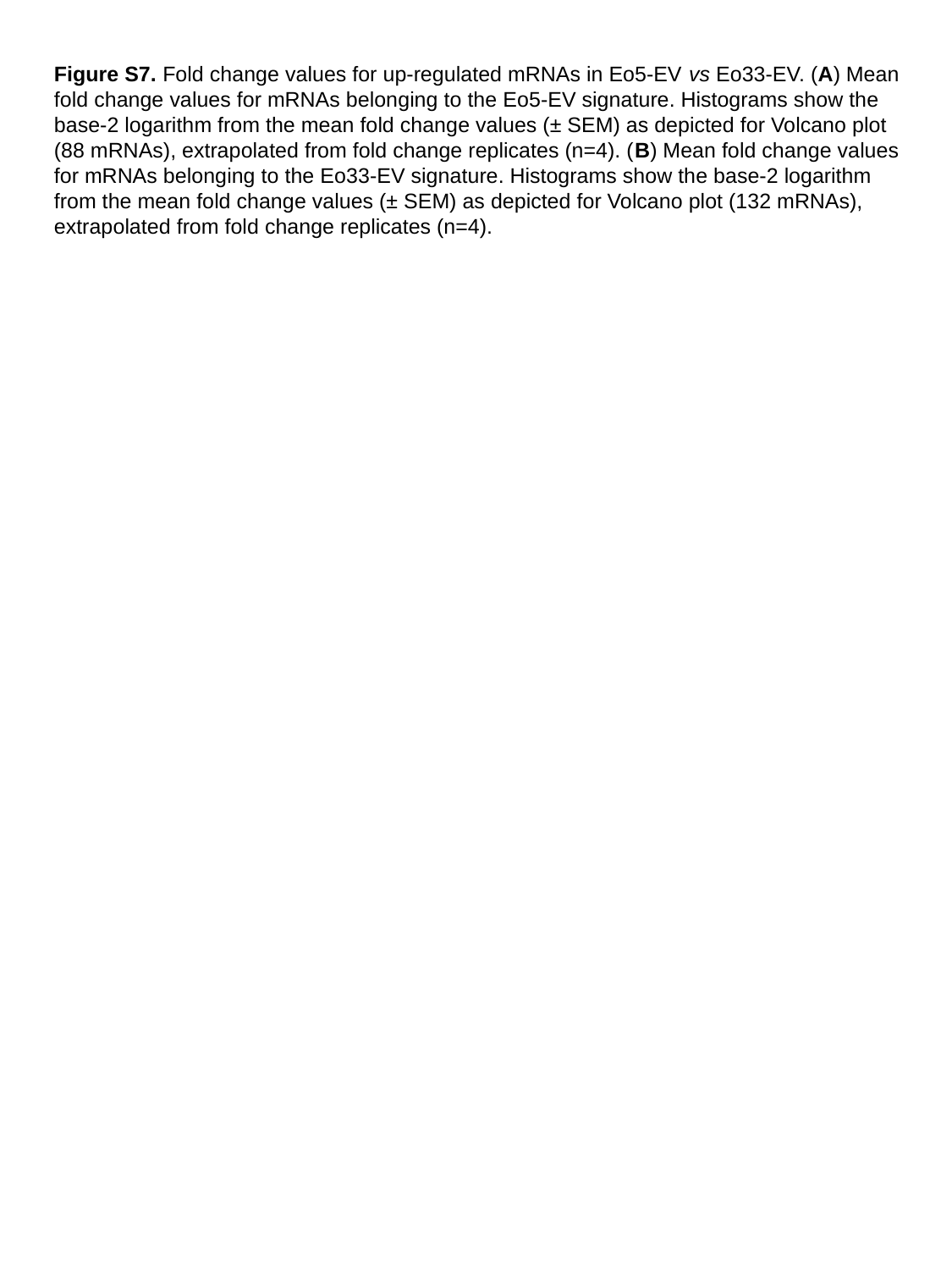

Figure S7. Fold change values for up-regulated mRNAs in Eo5-EV vs Eo33-EV. (A) Mean fold change values for mRNAs belonging to the Eo5-EV signature. Histograms show the base-2 logarithm from the mean fold change values (± SEM) as depicted for Volcano plot (88 mRNAs), extrapolated from fold change replicates (n=4). (B) Mean fold change values for mRNAs belonging to the Eo33-EV signature. Histograms show the base-2 logarithm from the mean fold change values (± SEM) as depicted for Volcano plot (132 mRNAs), extrapolated from fold change replicates (n=4).

## Slide 11
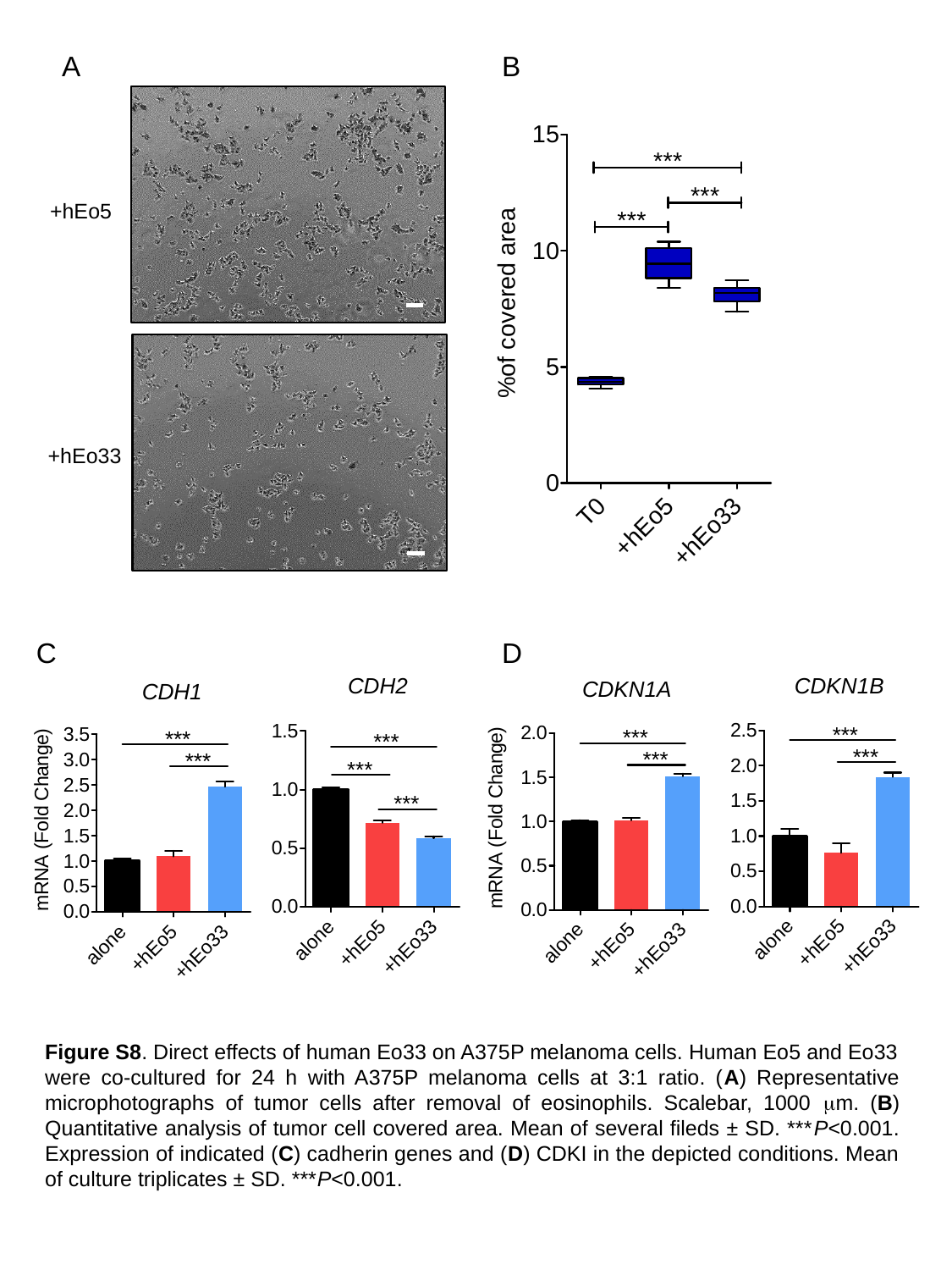

A
B
+hEo5
+hEo33
C
D
Figure S8. Direct effects of human Eo33 on A375P melanoma cells. Human Eo5 and Eo33 were co-cultured for 24 h with A375P melanoma cells at 3:1 ratio. (A) Representative microphotographs of tumor cells after removal of eosinophils. Scalebar, 1000 μm. (B) Quantitative analysis of tumor cell covered area. Mean of several fileds ± SD. ***P<0.001. Expression of indicated (C) cadherin genes and (D) CDKI in the depicted conditions. Mean of culture triplicates ± SD. ***P<0.001.

## Slide 12
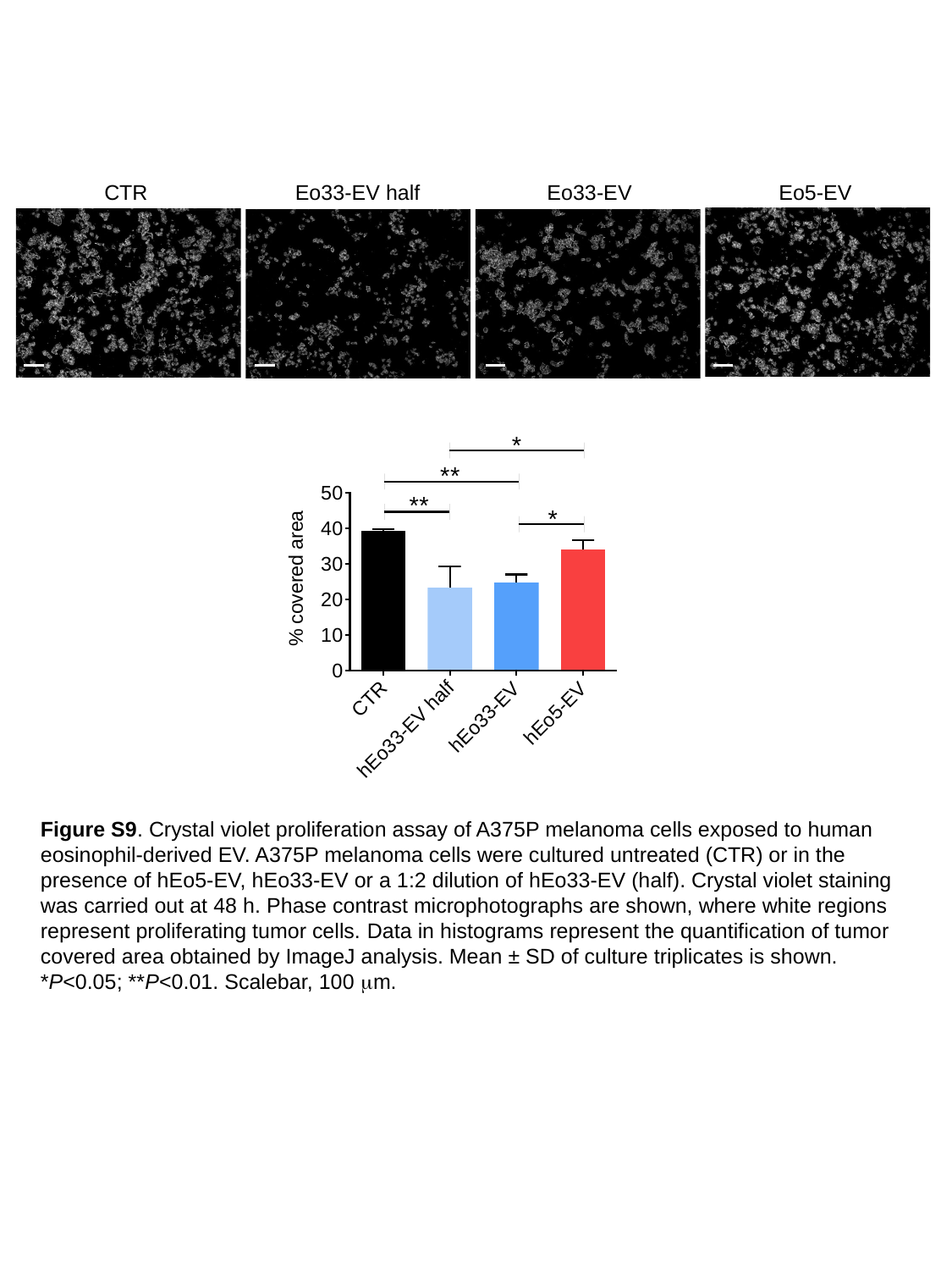

CTR
Eo33-EV half
Eo33-EV
Eo5-EV
Figure S9. Crystal violet proliferation assay of A375P melanoma cells exposed to human eosinophil-derived EV. A375P melanoma cells were cultured untreated (CTR) or in the presence of hEo5-EV, hEo33-EV or a 1:2 dilution of hEo33-EV (half). Crystal violet staining was carried out at 48 h. Phase contrast microphotographs are shown, where white regions represent proliferating tumor cells. Data in histograms represent the quantification of tumor covered area obtained by ImageJ analysis. Mean ± SD of culture triplicates is shown. *P<0.05; **P<0.01. Scalebar, 100 mm.
