# Supplementary material for: IL-33 stimulates the anticancer activities of eosinophils through extracellular vesicle-driven reprogramming of tumor cells
Source: J Exp Clin Cancer Res. 2024 Jul 27;43:209. doi: 10.1186/s13046-024-03129-1 (PMC11282757; doi:10.1186/s13046-024-03129-1)
Supplement: Supplementary file 2 — Supplementary Material 2. [file 13046_2024_3129_MOESM2_ESM.docx]

**Table S1. qRT-PCR primer pairs.**

| **Human** |  |  |
| --- | --- | --- |
| **Gene** | **Accession number** | **Primer sequence (Forward/Reverse, 5' --> 3')** |
| *CDKN1a* | NM_000389 | CCTCATCCCGTCCTTT |
|  |  | GTACCACCCAACAAGT |
| *CDKN1b* | NM_004064 | GATGCGTCCAGAAGCT |
|  |  | CGGCCCCAGTAAGTTG |
| *CDKN2a* | NM_058195 | CTCGTGCTGATGAGGA |
|  |  | GGTCGGCGCAGGCTCC |
| *CDKN2b* | NM_004936 | ACGGAGTCAACGGGAG |
|  |  | GGTCGGGTGAGGCAGG |
| *CDH1* | NM_004360.5 | AGCCCCGCCTTATGATTCTCTG |
|  |  | TGCCCCATTCGTTCAAGTAGTCAT |
| *CDH2* | NM_001308176.2 | ATGGGAAATGGAAACTTGATGGC |
|  |  | CAGTTGCTAAACTTCACTGAAAGG |
| *HPRT* | NM_000194.3 | TGACACTGGCAAAACAATGCA |
|  |  | GGTCCTTTTCACCAGCAAGCT |
| **Mouse** |  |  |
| **Gene** | **Accession number** | **Primer sequence (Forward/Reverse, 5' --> 3')** |
|  |  |  |
| *Cdkn1a* | NM_007669 | CCAGGCCAAGATGGTGTCTT |
|  |  | TGAGAAAGGATCAGCCATTGC |
| *Cdkn1b* | NM_009875 | GCGGTGCCTTTGGGTC |
|  |  | CGCTAACCCAGATTGT |
| *Cdkn2a* | NM_009877 | TGTTGAGGCTATCTTG |
|  |  | CGAATCTGCATTGAGC |
| *Cdkn2b* | NM_007670 | ATCCCAACGCACCGCT |
|  |  | AGTTGGGTTCGTGGAG |
| *Cdh1* | NM_009864.3 | AGACTTTGGTGTGGGTCAGG |
|  |  | CATGCTCAGCGTCTTCTCTG |
| *Cdh2* | NM_007664.5 | TGTGGAGGCTTCTGGTGAAAT |
|  |  | CTGAATTTCACATTGAGAAGGGGC |
| *Hprt* | NM_013556.2 | CTGGTGAAAAGGACCTCTCG |
|  |  | TGAAGTACTCATTATAGTCAAGGGCA |
|  |  |  |
